# Self-assembling 3D vessel-on-chip model with hiPSC-derived astrocytes

**DOI:** 10.1016/j.stemcr.2024.05.006

**Published:** 2024-06-13

**Authors:** Dennis M. Nahon, Marc Vila Cuenca, Francijna E. van den Hil, Michel Hu, Tessa de Korte, Jean-Philippe Frimat, Arn M.J.M. van den Maagdenberg, Christine L. Mummery, Valeria V. Orlova

**Affiliations:** 1 Department of Anatomy and Embryology, Leiden University Medical Centre, 2333ZA Leiden, the Netherlands; 2 Department of Clinical Genetics, Leiden University Medical Centre, 2333ZA Leiden, the Netherlands; 3 Department of Human Genetics, Leiden University Medical Centre, 2333ZA Leiden, the Netherlands; 4 Department of Neurology, Leiden University Medical Centre, 2333ZA Leiden, the Netherlands

**Keywords:** human induced pluripotent stem cells, organ-on-chip, vessel-on-chip, hiPSC-derived endothelial cells, hiPSC-ECs, hiPSC-derived astrocytes, hiPSC-Astro, blood-brain barrier, BBB, microfluidics

## Abstract

Functionality of the blood-brain barrier (BBB) relies on the interaction between endothelial cells (ECs), pericytes, and astrocytes to regulate molecule transport within the central nervous system. Most experimental models for the BBB rely on freshly isolated primary brain cells. Here, we explored human induced pluripotent stem cells (hiPSCs) as a cellular source for astrocytes in a 3D vessel-on-chip (VoC) model. Self-organized microvascular networks were formed by combining hiPSC-derived ECs, human brain vascular pericytes, and hiPSC-derived astrocytes within a fibrin hydrogel. The hiPSC-ECs and pericytes showed close interactions, but, somewhat unexpectedly, addition of astrocytes disrupted microvascular network formation. However, continuous fluid perfusion or activation of cyclic AMP (cAMP) signaling rescued the vascular organization and decreased vascular permeability. Nevertheless, astrocytes did not affect the expression of proteins related to junction formation, transport, or extracellular matrix, indicating that, despite other claims, hiPSC-derived ECs do not entirely acquire a BBB-like identity in the 3D VoC model.

## Highlights

• 3D vessel-on-chip (VoC) model with hiPSC-derived astrocytes (iAstros)
• Side-by-side comparison of iAstros to primary astrocytes in a 3D VoC model
• cAMP-pathway activation or fluid flow rescues 3D VoC model with iAstros

## Graphical abstract

**Figure:**
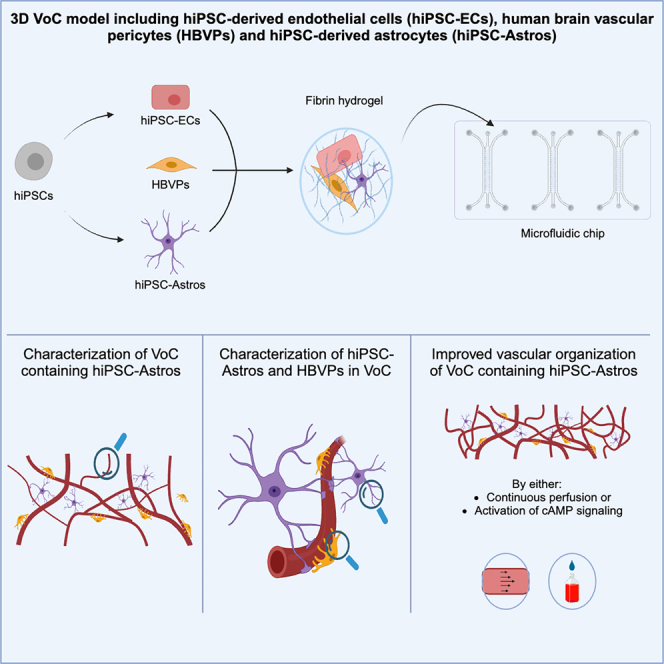

## Teaser

In this report, Nahon and colleagues present a self-assembling 3D VoC model composed of hiPSC-derived ECs, HBVPs, and hiPSC-derived astrocytes (iAstros). Microvascular networks formed and facilitated direct interactions between the incorporated cell types. Vascular network organization and permeability were rescued in a 3D VoC model with iAstros by cAMP-pathway activation or fluid flow.

## Introduction

The blood-brain barrier (BBB) is formed through direct interactions between endothelial cells (ECs), pericytes, and astrocytes in the central nervous system. BBB dysfunction is increasingly recognized as a contributor to multiple neurodegenerative diseases (Sweeney et al., 2018). This has led to many attempts to develop human *in vitro* models that recapitulate complex interactions between astrocytes and the vasculature (Hajal et al., 2021). Some aspects of the BBB *in vitro*, such as high transendothelial electrical resistance (TEER) and low permeability to soluble tracers, have been achieved by co-culturing primary brain microvascular- or cord blood-derived ECs with brain pericytes and astrocytes on a porous membrane (Boyer-Di Ponio et al., 2014; Cecchelli et al., 2014). However, primary brain cells are difficult to obtain and, even from commercial sources, show batch-to-batch variability. Human induced pluripotent stem cell (hiPSC)-derived brain microvascular ECs (BMECs) (Lippmann et al., 2012) have been widely used in engineering approaches for the BBB (Hajal et al., 2021). However, it later turned out that these actually resembled epithelial cells rather than ECs (Lu et al., 2021); this explained their abnormally high TEER values. More recently, alternative protocols to differentiate brain-like microvascular ECs that more closely resemble true ECs based on the expression of EC-specific markers and responses to proinflammatory stimuli have been developed (Gastfriend et al., 2021; Nishihara et al., 2020). These were useful for studying intrinsic defects in hiPSC-ECs derived from multiple sclerosis (MS) patient (Nishihara et al., 2022). In addition, several vessel-on-chip (VoC) models have been developed which combine the three most important cell types of the BBB: ECs, pericytes, and astrocytes. However, while they have some value, some of these models lack the direct heterotypic cell-cell and cell-matrix interactions typically seen *in vivo* (Maoz et al., 2018; Vatine et al., 2019). The models that do recapitulate these interactions using vasculogenesis or angiogenesis as starting points usually use primary cells including primary astrocytes (pAstros) (Campisi et al., 2018; Lee et al., 2020; Winkelman et al., 2022).

In this study, we aimed to develop an hiPSC-based 3D VoC model that integrates ECs from hiPSCs (hiPSC-ECs), human brain vascular pericytes (HBVPs), and hiPSC-derived astrocytes (hiPSC-Astros). We used our earlier protocol to differentiate ECs from hiPSCs (Orlova et al., 2014) and showed that astrocytes derived from hiPSCs could be integrated into the VoC and behaved much like human pAstros. hiPSC-Astros incorporated into a VoC triple culture containing both hiPSC-ECs and HBVPs self-assembled into microvascular networks in 3D with hiPSC-Astros and HBVPs assuming positions surrounding the vascular wall in the microfluidic chip. We also investigated two ways of improving microvascular network formation and organization in the VoC model with hiPSC-Astros: activating the cyclic AMP (cAMP) pathway or introducing continuous microfluidic flow. Despite improvements in relevant cell interactions and reproducibility, the model still falls short in reproducing a true BBB.

## Results

### 3D VoC integrating hiPSC-Astros

**Figure 1:**
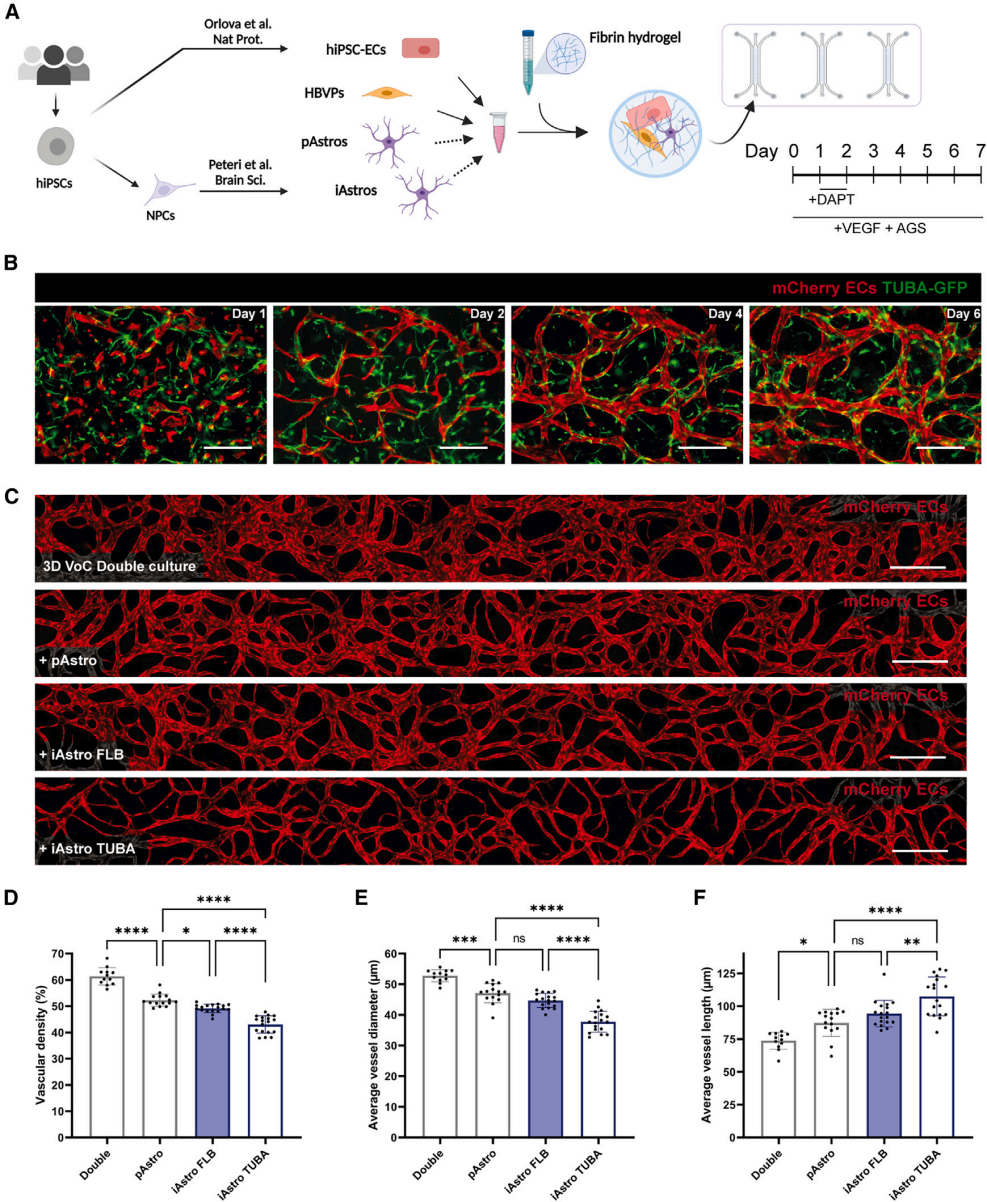
iAstros incorporated into 3D VoC model (A) Schematic of VoC protocol using hiPSC-ECs with HBVPs and pAstros or iAstros. (B) Representative immunofluorescence images of a 3D VoC triple culture containing hiPSC-ECs, HBVPs, and iAstros at day 1, 2, 4, and 6 showing hiPSC-mCherry ECs (red) and iAstros differentiated from the TUBA hiPSC line (green, TUBA-GFP). Scale bars: 250 μm. (C) Representative immunofluorescence images of microvascular networks in microfluidic chips on day 7 showing hiPSC-mCherry ECs (red). Images showing microvascular networks from a VoC double culture (hiPSC-ECs and HBVPs) or 3D VoC triple cultures including either pAstros or iAstros from two independent hiPSC lines (FLB or TUBA). Scale bars: 250 μm. (D–F) Quantification of full channel images of microvascular networks showing vascular density at endpoint day 7 showing (D), average vessel diameter (E), and average vessel length (F). Data are shown as mean ± SD. All conditions are *N* = 3, *n* = 12–18; three independent experiments with minimum of 3 microfluidic channels per experiment. One-way ANOVA with Sidak’s multiple comparison test. ^∗^*p* < 0.05, ^∗∗^*p* < 0.01, ^∗∗∗^*p* < 0.001, ^∗∗∗∗^*p* < 0.0001; ns, non-significant. See also Figures S1 and S2.

A 3D VoC model was established by combining hiPSC-ECs and HBVPs in a fibrin hydrogel and injecting the cell/gel mix into a commercially available AIM Biotech idenTx9 3D culture chip using a protocol described previously (Vila Cuenca et al., 2021) (Figure 1A). Either hiPSC-Astros or pAstros were included in the VoC model to mimic BBB cell combinations. First, hiPSC-derived neural progenitor cells (NPCs) were generated as previously described (Peteri et al., 2021) (Figure S1A). hiPSC-derived neural organoids contained neural rosette-like structures (Figure S1B), and more of these organoids could be formed in the expansion phase (Figure S1C). Proper patterning and differentiation were confirmed by the expression of the NPC markers SRY-box transcription factor 2 (SOX2) and paired box 6 (PAX6) and the forebrain marker forkhead box G1 (FOXG1) (Figure S1D). We then derived hiPSC-Astros in two different ways: one already published (Peteri et al., 2021) (iAstros) and the other using a commercially available kit (iSCT Astros) (Figure S1A). iAstros and iSCT Astros showed comparable expression of key astrocyte markers, such as glial fibrillary acidic protein (GFAP), fatty acid binding protein 7 (FABP7), S100 calcium binding protein B (S100β), vimentin, and solute carrier family 1 member 3 (SLC1A3/GLAST) (Figures S1E and S1F). In addition, iAstros showed increased intracellular Ca^2+^ release upon stimulation with adenosine triphosphate (ATP) (3 μM and 300 μM) (Figure S1G and S1H) and efficient uptake of the neurotransmitter glutamate (Figure S1F), confirming their functionality.

A triple culture of hiPSC-ECs derived from a control mCherry reporter hiPSC line, HBVPs, and iAstros derived from a control hiPSC line with an α-tubulin-mEGFP reporter (AISC0012, TUBA) (Roberts et al., 2017) was monitored from day 1 to day 6 (Figure 1B). hiPSC-ECs self-organized into interconnected microvascular networks within 2–3 days, with fully lumenized structures by day 7 (Figure 1B). In addition, iAstros localized in the extravascular space, interacting directly with the developing microvascular network (Figure 1B). The development of a robust VoC triple culture model was confirmed by similarly including iSCT Astros from two independent hiPSC lines (FLB or TUBA) (Figure S2). The remaining experiments were performed using iAstros, and not iSCT Astros, as iAstros can be cryopreserved at the endpoint of differentiation and used as a convenient cell source for 3D VoC triple culture setups. The vascular beds thus established were compared between VoC double cultures (only containing hiPSC-ECs and HBVPs) and VoC triple cultures including either pAstros or iAstros from two independent hiPSC lines (FLB or TUBA). All cell combinations formed an interconnected microvascular network by day 7 in a highly reproducible manner across independent experiments (Figures 1C–1F). Quantification of vessel parameters showed that inclusion of any astrocyte source in our VoC model reduced vascular density (%, Figure 1D) and average vessel diameter (μm, Figure 1E) and increased average vessel length (μm, Figure 1F) relative to the double cultures. Significant differences were observed in vessel parameters between pAstro and iAstro triple cultures depending on the hiPSC line used. This is in line with batch-to-batch variability of pAstros previously described (Hajal et al., 2022). Specifically, incorporating iAstros from either the FLB or TUBA hiPSC line into our VoC model resulted in a significant decrease in vascular density compared to pAstros (Figure 1D). However, only iAstros from the TUBA hiPSC line caused a significant decrease in average vessel diameter and increase in average vessel length (Figures 1D–1F).

### Characterization of astrocytes and HVBPs in the 3D VoC model

**Figure 2:**
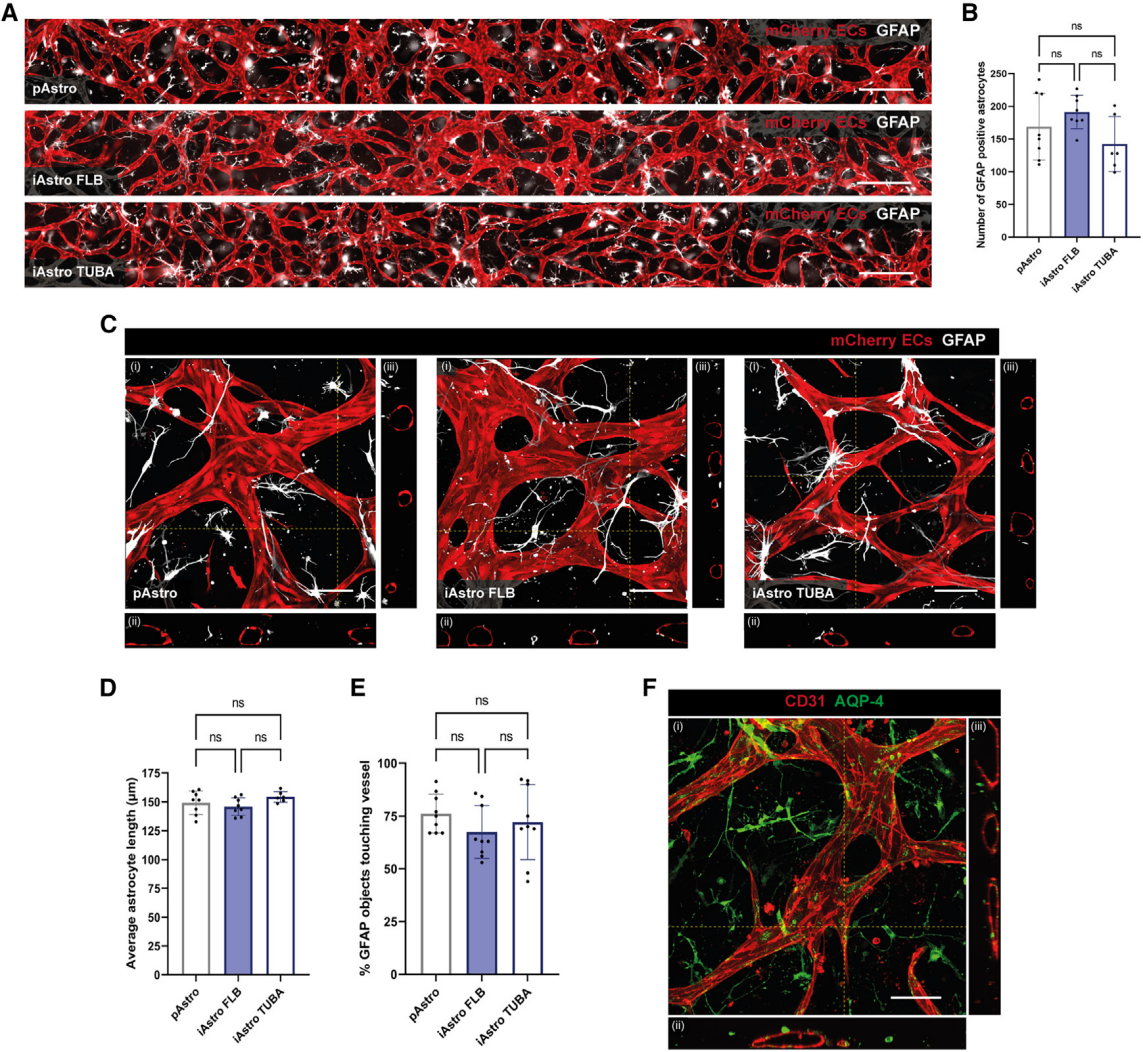
Comparable structural properties of primary and hiPSC-Astros in a 3D VoC model (A) Representative immunofluorescence images of microvascular networks in microfluidic chips on day 7 showing hiPSC-mCherry ECs (red) and astrocytes (silver; GFAP). Images showing VoC triple cultures of hiPSC-ECs with HBVPs and pAstros or iAstros from FLB or TUBA hiPSC line. Scale bars: 500 μm. (B) Quantification of astrocytes in VoC triple cultures showing number of GFAP-positive astrocytes in ±80% of full microfluidic channel. ns, non-significant. (C) Representative immunofluorescence confocal images of microvascular networks in microfluidic chips showing hiPSC-mCherry ECs (red) and astrocytes (silver; GFAP). Images displaying xyz (i), xy (ii), and yz cross-sectional perspectives (iii). Images showing VoC triple cultures of hiPSC-ECs with HBVPs and pAstros or iAstros from the FLB or TUBA hiPSC lines. Scale bars: 100 μm. (D and E) Quantification of astrocytes in VoC model showing average astrocyte length (D) and % of GFAP objects touching the microvascular network (E). Data are shown as mean ± SD. For (B) and (D) *N* = 3, n = 6–8; three independent experiments with a minimum of two microfluidic channels per experiment. For (E) *N* = 3, *n* = 3; three independent experiments, one microfluidic channel per experiment with three regions of interest (ROIs) per channel. One-way ANOVA with Tukey’s multiple comparison. ns, non-significant. (F) Representative immunofluorescence confocal image of microvascular network in microfluidic chips showing ECs (red; CD31) and astrocytes (green; Aqp4) in a VoC triple culture of hiPSC-ECs with HBVPs and iAstros from the FLB hiPSC line. Images displaying xyz (i), xy (ii), and yz cross-sectional perspectives (iii). Scale bar: 100 μm. See also Video S1.

We next examined astrocyte morphology and interaction with hiPSC-EC in microvascular networks in the VoC model. Both pAstros and iAstros from either the FLB or TUBA hiPSC lines stained positively for GFAP and showed uniform distribution through the entire microfluidic channel with no significant differences in the total number of GFAP-positive cells (Figures 2A and 2B). Astrocytes in all conditions showed a distinct stellate morphology and were positioned closely to the abluminal side of the microvascular networks (Figure 2C, Video S1). No significant differences in the average astrocyte length (μm, Figure 2D) or the percentage of astrocytes associated with the microvascular network (%, Figure 2E) were observed between the different sources of astrocytes upon quantification of the confocal images. In addition, iAstros stained positively for the astrocyte-specific water channel aquaporin 4 (AQP4) (Figure 2F), although the staining was distributed across the plasma membrane without polarized expression in the astrocyte endfoot.

**Figure 3:**
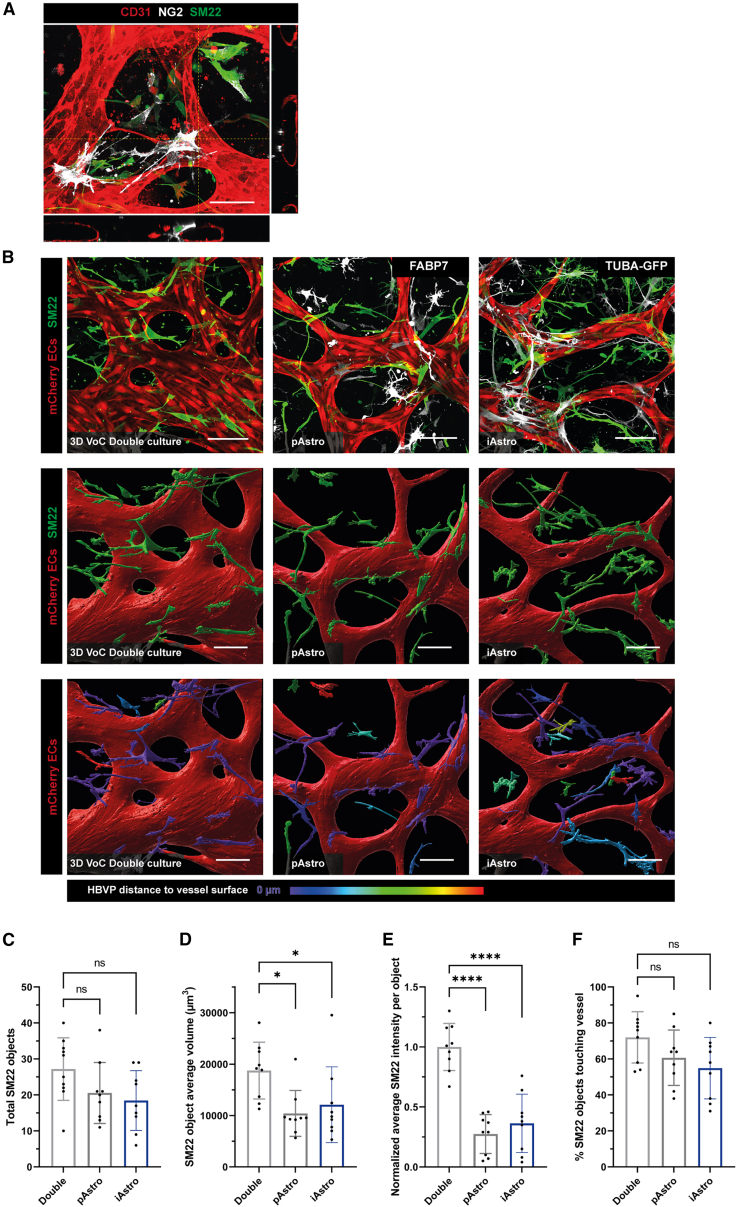
Comparable structural properties of HBVPs in a 3D VoC model including astrocytes (A) Representative immunofluorescence confocal image of microvascular network in microfluidic chips showing ECs (red; CD31) and HBVPs (silver; NG2, green; SM22) in a VoC double culture of hiPSC-ECs with HBVPs. Images displaying xyz (i), xy (ii), and yz cross-sectional perspectives (iii). Scale bar: 100 μm. (B) Representative immunofluorescence confocal images of microvascular networks in microfluidic chips on day 7 showing hiPSC-mCherry ECs (red), HBVPs (green; SM22), and pAstros (silver, FABP7) or iAstros from the TUBA hiPSC line (silver, TUBA-GFP), surface-rendered images and color-coded images of HBVPs distance to the vessel surface. Images showing VoC double cultures (hiPSC-ECs with HBVPs) and VoC triple cultures including either pAstros or iAstros from the TUBA hiPSC line. Scale bars: 100 μm. (C–F) Quantification of HBVPs in VoC double and triple cultures containing iAstros from the TUBA hiPSC line showing number of SM22-positive objects (C), average SM22 object volume (D), normalized average SM22 intensity per object (E), and percentage of SM22 objects touching the microvascular network (F). Data are shown as mean ± SD. For (C–F) *N* = 3, n = 9–10; three independent experiments, one microfluidic channel per experiment with three or four ROIs per channel. Scale bars: 100 μm. One-way ANOVA with Sidak’s multiple comparison test. ^∗^*p* < 0.05, ^∗∗∗∗^*p* < 0.0001; ns, non-significant. See also Video S1.

Video S1. 3D confocal reconstruction of EC-HBVP and ECiAstro interactions, related to Figure 2 and Figure 3

We confirmed the identity of the HBVPs in the 3D VoC culture by overlaying immunostaining for the pericyte marker neuron-glial antigen 2 (NG2) and contractile marker smooth muscle protein 22 (SM22) (Figure 3A). We previously demonstrated that SM22 is indicative of heterotypic cell-cell contact-induced HBVP cell maturation in VoC cultures (Vila Cuenca et al., 2021) and confirmed similar cell-cell interactions in our current VoC setup (Video S1). By using SM22, we therefore investigated whether HBVPs were affected by the addition of astrocytes. Staining of the VoC triple cultures showed SM22 in both HBVPs and astrocytes. To distinguish HBVPs from astrocytes, we used the fluorescently tagged TUBA-GFP iAstros and co-stained the VoC model including pAstros with the glial marker FABP7. Surface rendering of confocal images and identification of SM22+ HBVPs revealed similar numbers of HBVPs in the VoC double cultures and VoC triple cultures including pAstro or iAstros (Figures 3B and 3C). SM22+ HBVPs in the microvascular network had a reduced average volume and normalized average SM22 intensity in the VoC triple cultures with astrocytes (μm^3^, Figures 3D and 3E). No significant differences were observed in the direct interaction of the HBVPs with the microvascular network between the different conditions (Figures 3B; %, 3F).

### Continuous perfusion or activation of cAMP signaling improves vascular organization and reduces permeability in the 3D VoC model

**Figure 4:**
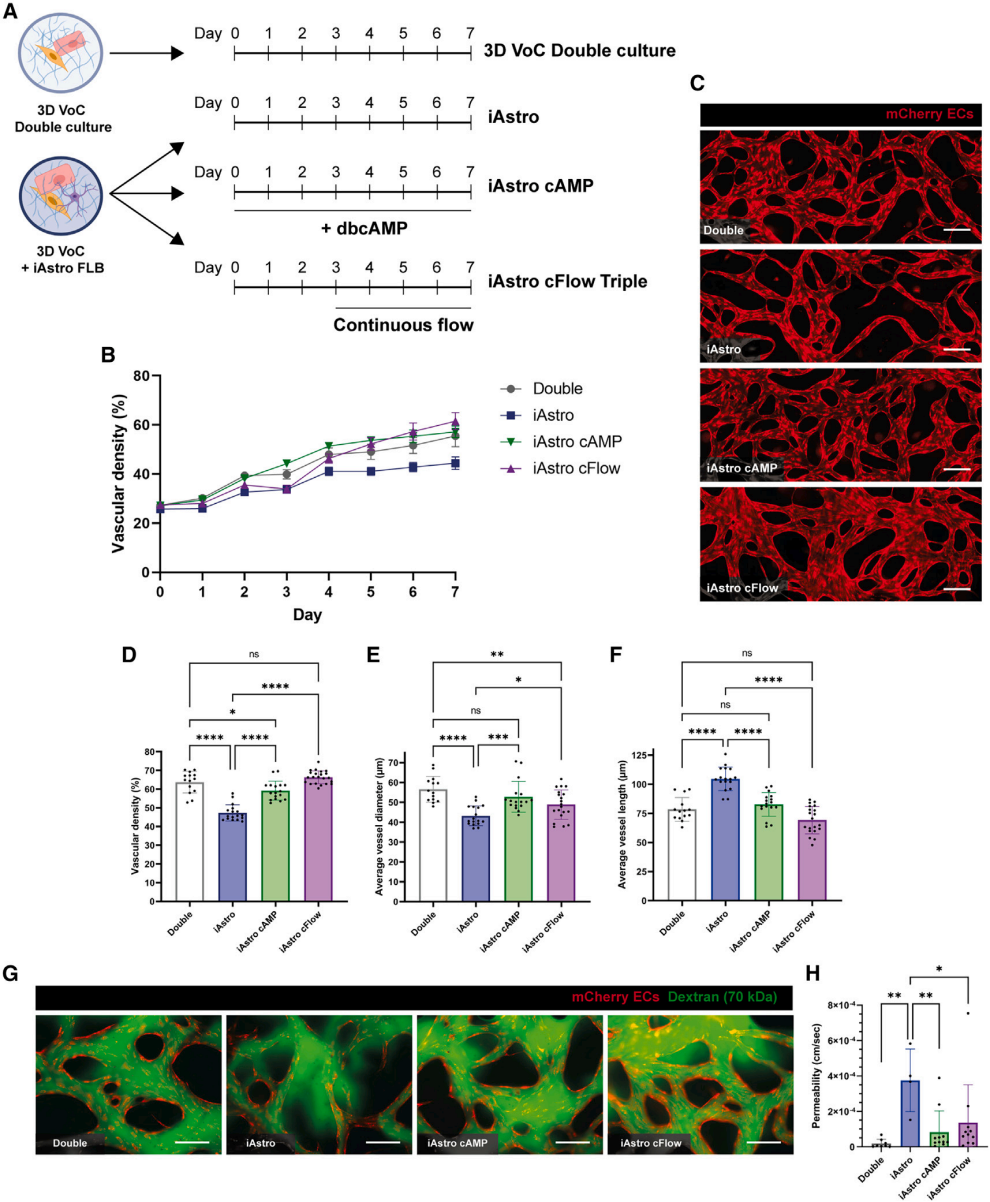
Improved microvascular network of 3D VoC triple culture including hiPSC-Astros upon activation of cAMP signaling or application of continuous perfusion (A) Schematic of experimental setup for improving microvascular network formation of VoC triple cultures containing hiPSC-ECs, HBVPs, and iAstros from the FLB iPSC line. In the iAstro cAMP condition, the medium was daily supplemented with 250 μM dbcAMP to activate cAMP signaling. In the iAstro continuous flow (cFlow) condition, the VoC triple culture was subjected to continuous flow from day 3 onwards. (B) Quantification of vessel density over time from daily images of hiPSC-mCherry ECs for the four VoC culture conditions. (C) Representative immunofluorescence images of microvascular networks from the four VoC culture conditions at day 7 showing hiPSC-mCherry ECs (red). iAstro conditions are triple cultures containing iAstros from the FLB hiPSC line. Scale bars: 200 μm. (D–F) Quantification of microvascular networks showing vascular density at endpoint day 7 (D), average vessel diameter (E), and average vessel length (F). Data are shown as mean ± SD. Data shown are N = 3–4, *n* = 14–22; three or four independent experiments with a minimum of 3 microfluidic channels per experiment. iAstro conditions are triple cultures containing iAstros from the FLB hiPSC line. (G) Representative immunofluorescence images of microvascular networks (red; hiPSC-mCherry ECs) perfused with 70 kDa FITC-dextran (green) on day 7, 30 s after start of perfusion. Images show VoC double cultures (hiPSC-ECs with HBVPs) and VoC triple cultures also containing iAstros from the FLB hiPSC line. Scale bars: 200 μm. (H) Quantification of permeability coefficient for the four VoC culture conditions at endpoint day 7 from N = 3–4, n = 4–11; three or four independent experiments with one to six microfluidic channels per experiment. In the iAstro conditions, data are pulled from triple cultures containing both iAstros from the FLB and the TUBA hiPSC lines. One-way ANOVA with Sidak’s multiple comparison test. ^∗^*p* < 0.05, ^∗∗^*p* < 0.01 ^∗∗∗^*p* < 0.001, ^∗∗∗∗^*p* < 0.0001; ns, non-significant. See also Figures S3 and S4.

In addition to reduced microvascular network density and diameter and SM22 volume and intensity, more detailed examination showed local disruptions in the EC layer in both pAstro and iAstro triple culture conditions, although not in the double culture condition (Figure S3A). To improve the triple culture VoC model, we investigated whether culture conditions, postulated to modulate maturation of astrocytes, or microfluidic flow might improve organization of the microvascular networks (Figure 4A). Specifically, we investigated the effect of activation of the cAMP pathway since it has not only been reported to improve astrocyte maturity and immune response (Reuschlein et al., 2019; Zhou et al., 2019) but also shown to be protective for endothelial integrity and barrier function in the BBB (Viña et al., 2021). The influence of continuous flow was studied since mechanical forces resulting from luminal flow through blood vessels are known to promote EC survival, migration, and proliferation (Campinho et al., 2020). VoC triple cultures including iAstros were thus either supplemented daily with dibutytyl cAMP (dbcAMP) (250 μM), the cell membrane-permeable analog of cAMP, or subjected to continuous flow from day 3 till day 7 (Figure 4A). Both dbcAMP addition and continuous flow in VoC triple cultures including iAstros increased vascular density over time (%, Figure 4B). This was most evident on day 7, where both the cAMP and continuous flow conditions significantly increased vascular density and average vessel diameter and decreased average vessel length in comparison to standard VoC triple cultures (Figure 4C; %, 4D; μm, 4E; μm, 4F).

We subsequently investigated whether dbcAMP or continuous perfusion affected microvascular network formation through increased proliferation or through increased presence of matrix metalloproteinase-2 (MMP2), as was shown in a recent study of a VoC containing primary brain ECs, HBVPs, and pAstros (Zhang et al., 2022). Proliferation was quantified by 5-ethynyl-2′-deoxyuridine (EdU) pulse experiments on day 4 of culture. Continuous flow in the VoC model with iAstros increased proliferation compared to the control VoC cultures with or without (dbcAMP-treated) iAstros (Figures S3B and S3C). Co-staining of microfluidic channels with the EC-specific transcription factor SOX17 revealed that most of proliferating cells were hiPSC-ECs (Figures S3D and S3E). Quantitative real-time PCR (real-time qPCR) of VoC cultures at endpoint day 7 confirmed the increase in MMP2 in the iAstro VoC triple culture under continuous flow conditions (Figure S3F).

We next perfused VoC cultures with fluorescein isothiocyanate (FITC)-dextran (70 kDa) to investigate local vascular barrier integrity and quantified the permeability coefficient of the four VoC triple culture conditions (Figures 4G and 4H; cm/s). Both addition of dbcAMP and application of continuous flow improved local vascular integrity and significantly decreased the permeability coefficient in iAstro VoC triple cultures (Figure S3G).

Finally, we explored the influence of the different VoC conditions on the expression of BBB-related markers. Real-time qPCR of VoC culture conditions at endpoint day 7 was somewhat variable between experiments and did not show a significant increase in most of the BBB-related genes investigated in triple culture conditions (Figure S4A). Integration of astrocytes had no effect on the expression of adherens and tight junction markers, such as vascular endothelial cadherin (VEC), zonula occludens-1 (ZO1), and claudin-5 (CLDN5) by real-time qPCR (Figure S4A) and by immunohistochemistry (Figure S4B). Some upregulation of transport-related genes, such as solute carrier family 2 member 1 (SLC2A1) and p-glycoproteïne (PGP), and extracellular matrix (ECM)-related gene collagen type IV alpha 1 chain (COL4A1) was observed by real-time qPCR (Figure S4A) under continuous flow, but this was less evident by immunohistochemistry (Figures S4C and S4D).

## Discussion

In this study, we described the generation of 3D microvascular networks containing hiPSC-ECs, HBVPs, and hiPSC-Astros. We showed that these triple cultures develop interconnected, lumenized, perfusable networks, with direct interaction between the incorporated cell types. No apparent differences were observed in the morphology and expression of reactive markers in astrocytes, in any of the culture conditions tested in this study. Although the number of HBVPs and their interaction with the microvascular network were similar between culture conditions, the average volume and expression of the contractile marker SM22 differed between HBVPs in double and triple cultures. This could indicate that both pAstros and hiPSC-Astros affect the maturity and contractile phenotype of the HBVPs in our system. Interestingly, we observed decreased vascular density and diameter of the vessels upon adding pAstros or iAstros, similar to earlier studies also using pAstros (Campisi et al., 2018; Lee et al., 2020). In addition, we showed that appropriate culture medium and fluidic flow are important in the formation and stability of microvascular networks containing hiPSC-ECs, HBVPs, and hiPSC-Astros. We demonstrated that the addition of dbcAMP improved microvascular network formation and organization and vascular permeability in the VoC model that included iAstros. This is in line with previous reports demonstrating the importance of cAMP signaling in astrocyte and EC function (Ishizaki et al., 2003; McRae et al., 2018; Reuschlein et al., 2019; Viña et al., 2021; Zhou et al., 2019). Interestingly, EC proliferation was not increased by adding dbcAMP, although we cannot exclude timing of the EdU experiment being sub-optimal. Future studies will need to clarify the exact mechanism by which dbcAMP acts in this 3D model. We also showed that microfluidic flow promoted EC proliferation and improved stability of the triple culture microvascular networks. In addition, we observed increased MMP2 expression as shown previously (Zhang et al., 2022), even though this earlier study investigated the effect of interstitial flow in the first stages of vasculogenesis while we only applied flow when a lumenized microvascular network had already formed, primarily resulting in luminal shear stress.

Although we observed interaction between various cell types in our system, adding iAstros to the VoC model did not consistently increase BBB-related markers in hiPSC-ECs. This was independent of whether iAstros were from FLB or TUBA hiPSC lines. Improvements in the metabolic environment, adding other relevant small molecules or cytokines or altering the transcriptional regulation of hiPSC-ECs using transcription factors, will be required for better recapitulation of the BBB (Lu et al., 2021). Nevertheless, the model does represent an opportunity to be entirely hiPSC based, by replacing HBVPs with hiPSC-derived smooth muscle cells (Vila Cuenca et al., 2021), which would allow investigation of cell type-specific contributions in disease phenotypes as each could be replaced by an (isogenic) mutant variant.

In summary, we established a 3D VoC model containing hiPSC-ECs, HBVPs, and hiPSC-Astros mimicking the direct cell-cell interactions seen *in vivo*. We demonstrated that hiPSC-Astros perform similarly to pAstros and can thus be used as an alternative to primary brain tissue. We demonstrated that our model can recapitulate the complex interactions between multiple cell types within the BBB, crucial for studying diseases like cerebral amyloid angiopathy, other forms of vascular dementia, and conditions related to neuroinflammation. However, we noted that the extent to which hiPSC-Astros influence microvascular network formation can be hiPSC line dependent. Increasing cAMP signaling or introducing continuous flow improved microvascular networks containing hiPSC-Astros. Nevertheless, culture conditions will need further refinement before hiPSC models resemble the human BBB sufficiently for widespread use in studying neurodegenerative disorders and screening for new therapeutic interventions.

## Experimental procedures

### Resource availability

#### Lead contact

Requests for further information or more detailed protocols should be directed to and will be fulfilled by the corresponding author, Valeria V. Orlova (v.orlova@lumc.nl).

#### Materials availability

This study did not generate new unique reagents.

#### Data and code availability

Data will be shared with the research community upon request. No code or standardized datasets were generated.

### hiPSC lines

Research on hiPSC was approved by the medical ethical committee at Leiden University Medical Center, the Netherlands. A detailed list of the hiPSC lines and batches used for each experiment is provided in Table S1.

### VoC setup and culture

Cells were prepared prior to incorporation in VoCs as described in supplemental experimental procedures. Microvascular networks inside microfluidic chips were generated as previously described (Orlova et al., 2022; Vila Cuenca et al., 2021) with minor modifications. Microfluidic chips with one middle gel channel flanked by two media channels (AIM Biotech, idenTx9 chip) were used. Cells were resuspended in endothelial cell growth medium 2 (EGM-2) medium supplemented with thrombin (4 U/mL, Sigma, T4648) at 15 × 10^6^ cells/mL for hiPSC-ECs, 3 × 10^6^ cells/mL for HBVPs, and 7.5 × 10^6^ cells/mL for astrocytes (5:1:2.5 ratio, respectively). Three astrocyte cell suspensions were tested in combination with hiPSC-ECs and HBVPs: (1) pAstros, (2) iAstros, and (3) iSCT Astros. The cell suspensions were mixed with an equal volume of fibrinogen solution (6 mg/mL, final concentration 3 mg/mL, Sigma, 8630) and immediately injected into the gel channel of the microfluidic chip (3 gel channels per cell/fibrin mix and 15 μL per gel channel). Chips were incubated for 15 min at room-temperature (RT) before adding EGM-2 supplemented with 50 ng/mL vascular endothelial growth factor (VEGF) and 1% astrocyte growth supplement (AGS; Sciencell, 1852) to the media channels. The microfluidic chips were refreshed every 24 h with EGM-2 supplemented with VEGF (50 ng/mL) and 1% AGS. Refreshing was done by application of a hydrostatic pressure over the medium channel by adding 100 μL medium to the right media ports and 50 μL medium to the left media ports. On day 1, γ-secretase inhibitor N-[N-(3,5-difluorophenacetyl)-*l*-alanyl]-*s*-phenylglycinet-butyl ester (DAPT, 10 μM, Sigma, D5942) was added for 24 h. For cAMP condition, the media were additionally supplemented with 250 μM dbcAMP (Sigma, D0627) for the entire duration of culture. For the continuous perfusion condition, the microfluidic chips were placed on an interval rocker platform (Perfusion Rocker, MIMETAS) set at a 5-degree inclination and 8 min cycle time from day 3 onwards.

### Statistical analysis

Statistical analyses were performed using GraphPad Prism 9 software. Normality of the data was evaluated by the Shapiro-wilk test. One-way ANOVA with Tukey’s multiple comparison test or Sidak’s multiple comparison test was used for comparing multiple groups. Detailed statistics are indicated in each figure legend. The data are reported as mean ± SD.

## Supplemental information

Document S1. Figures S1–S4, Tables S1–S3, and supplemental experimental procedures

Document S2. Article plus supplemental information
