# Supplementary material for: Self-assembling 3D vessel-on-chip model with hiPSC-derived astrocytes
Source: Stem Cell Reports. 2024 Jun 13;19(7):946–56. doi: 10.1016/j.stemcr.2024.05.006 (PMC11252484; doi:10.1016/j.stemcr.2024.05.006)
Supplement: Document S1. Figures S1–S4, Tables S1–S3, and supplemental experimental procedures [file mmc1.pdf]

Stem Cell Reports, Volume *19*

Supplemental Information

Self-assembling 3D vessel-on-chip model with hiPSC-derived astrocytes

Dennis M. Nahon, Marc Vila Cuenca, Francijna E. van den Hil, Michel Hu, Tessa de Korte, Jean-Philippe Frimat, Arn M.J.M. van den Maagdenberg, Christine L. Mummery, and Valeria V. Orlova

Inventory of Supplemental information

Supplemental figures and legends:
Figure S1. Related to Figure 1. Characterization of hiPSC-astrocytes.

Figure S2. Related to Figure 1. iSCT Astros incorporated into 3D VoC model.

Figure S3. Related to Figure 4. Microvascular network integrity in VoC conditions and increased proliferation and increased expression of MMP2 upon continuous flow in 3D VoC triple cultures including astrocytes.

Figure S4. Related to Figure 4. Assessment of blood-brain barrier properties in 3D VoC cultures.

Supplemental Table:
Supplemental Table 1. List of hiPSC lines and batches used per experiment.
Supplemental Table 2. List of antibodies for immunofluorescence.
Supplemental Table 3. List of primers for qRT-PCR.

Supplementary Video:
Video S1. Related to Figure 2 and Figure 3. 3D confocal reconstruction of EC-HBVP and EC-iAstro interactions

Supplemental Experimental Procedures

Supplemental References

SUPPLEMENTAL FIGURE 1.

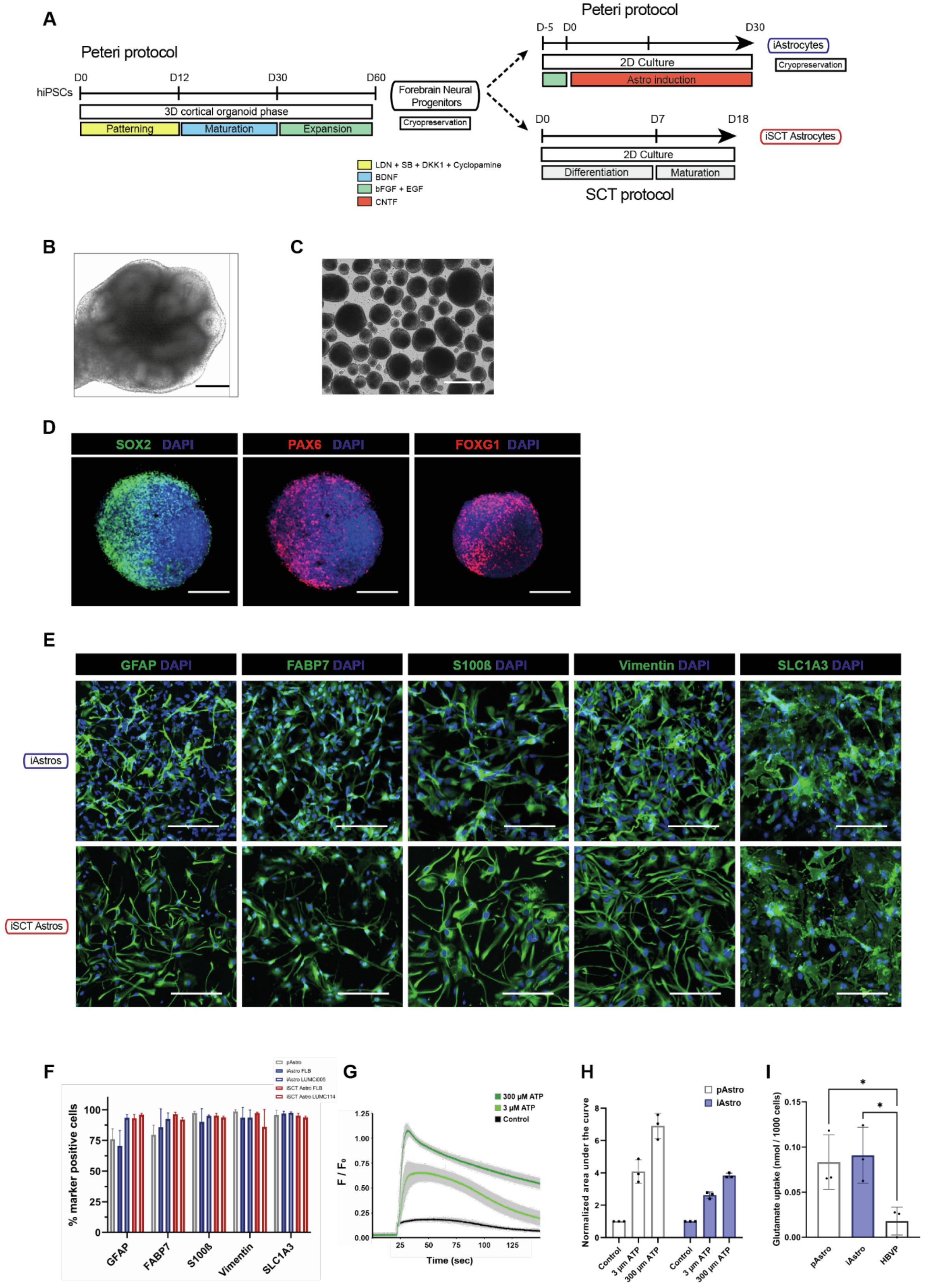

Figure S1. Related to Figure 1. Characterization of hiPSC-astrocytes.

(A) Schematic of ‘Peteri’ protocol (iAstro) and ‘SCT’ protocol (iSCT Astro) to generate forebrain patterned astrocytes from hiPSCs. (B) Representative brightfield image of a regionalized hiPSC-derived neural organoids on day 30. Scale bar: 250 µm. (C) Representative brightfield image of hiPSC-derived neural organoids on day 60. Scale bar: 500 µm. (D) Representative immunofluorescence images of hiPSC-derived neural organoids (day 72) differentiated from the FLB hiPSC line stained for SOX2, PAX6 or FOXG1. Scale bars: 100 µm. (E) Representative immunofluorescence images of iAstros and iSCT Astros stained for GFAP, FABP7, S100ß, Vimentin and GLAST. Scale bars; 200 µm. (F) Image based quantification of the percentage of marker positive cells. Data are shown as mean ± SD. pAstro, iAstro FLB and LUMCi005 are N = 3. iSCT Astro FLB and LUMC114 are N = 2. (G) Assessment of intracellular Ca^{2+} release in iAstros. Representative traces of normalized average fluorescence intensity (F/F0) in iAstros. Astrocytes were either stimulated by automated addition of plain NS medium (black) or NS medium supplemented with 3 µM (light green) or 300 µM (dark green) ATP. Data are shown as mean ± SD of N = 3; iAstros from one hiPSC line (FLB), one differentiation, in three independent experiments. (H) Quantification of intracellular Ca^{2+} release; normalized area under the curve, for pAstros and iAstros. Data shown as mean ± SD of N = 3; iAstros from one hiPSC line (FLB), three differentiations, in three independent experiments. (I) Quantified glutamate uptake normalized to the number of cells (nmol/1000 cells) for pAstros, iAstros and HBVP. Data shown as mean ± SD of N = 3, n = 6; one batch of pAstros and HBVPs and iAstros from one hiPSC line (FLB), three differentiations, in three independent experiments. One-way ANOVA with Tukey’s multiple comparison. $*p < 0.05$; ns, non-significant.

SUPPLEMENTAL FIGURE 2.

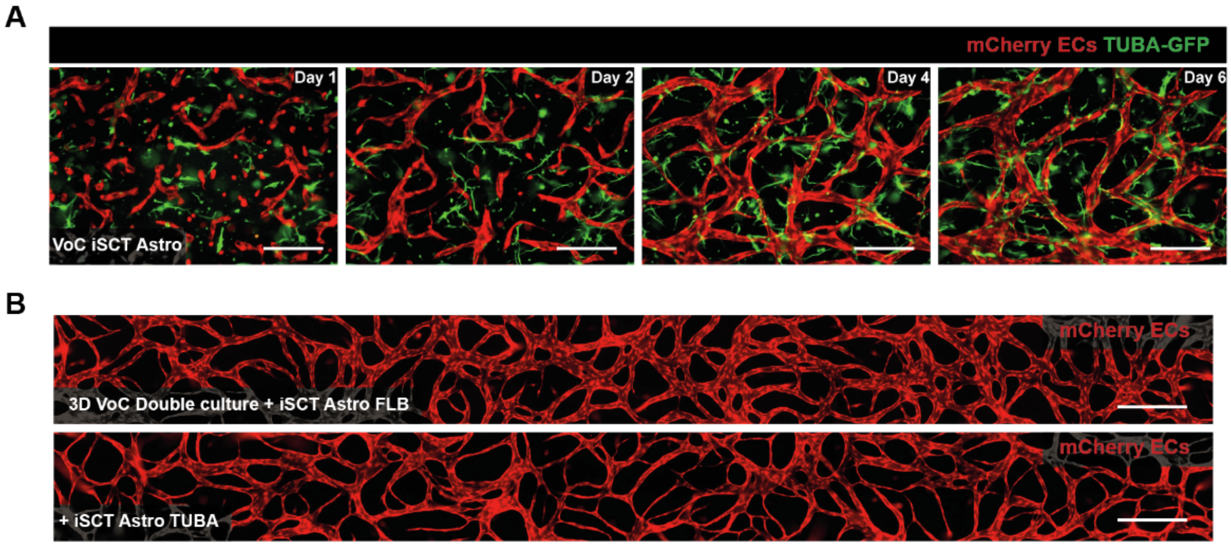

Figure S2. Related to Figure 1. iSCT Astros incorporated into 3D VoC model.
(A) Representative immunofluorescence images from day 1, 2, 4 and 6 showing hiPSC-mCherry ECs (red) and hiPSC-TUBA astrocytes (green, TUBA-GFP) from the 'SCT' protocol (iSCT Astro) in VoC triple cultures. Scale bars: 250 μm. (B) Representative immunofluorescence images of microvascular networks in microfluidic chips on day 7 showing hiPSC-mCherry ECs (red). Images showing microvascular networks from 3D VoC triple cultures including iSCT Astros from two independent hiPSC lines (FLB or TUBA). Scale bars: 250 μm.

SUPPLEMENTAL FIGURE 3.

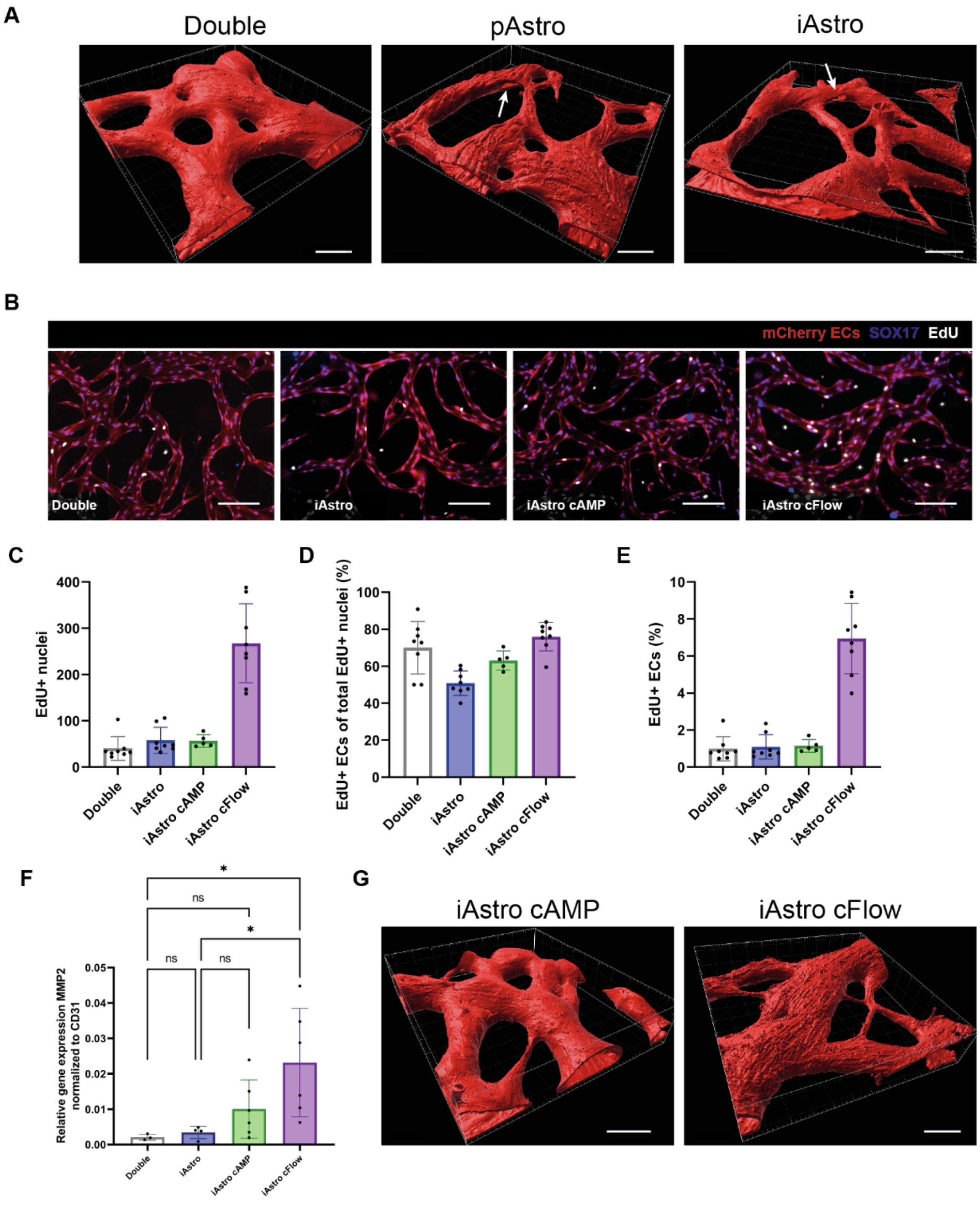

Figure S3. Related to Figure 4. Microvascular network integrity, increased proliferation and MMP2 expression upon continuous flow in 3D VoC triple cultures with astrocytes.

(A) Representative immunofluorescence confocal surface rendered images of microvascular networks in microfluidic chips of VoC cultures on day 7 showing hiPSC-mCherry ECs (red). Scale bars: 100 µm.

(B) Representative images showing proliferating cells (silver; EdU) and ECs (red and green; mCherry-ECs and SOX17 respectively) in VoC double cultures (hiPSC-ECs with HBVPs) and VoC triple cultures with iAstros from the FLB hiPSC line. In the iAstro cAMP condition, medium was daily supplemented with 250 µM dbcAMP and in iAstro continuous flow (cFlow) condition, microfluidic chips were continuously perfused from day 3 onwards. Microfluidic channels were fixed and stained at day 4. Scale bars: 200 µm. (C-E) Quantification of proliferation showing the total number of EdU positive nuclei (C), percentage of

proliferating cells which are ECs ((EdU+ SOX17+)/(EdU+)*100) (D) and percentage of ECs which are proliferating ((EdU+ SOX17+)/(SOX17+)*100) (E). Data shown as mean ± SD of N = 2, n = 8; two independent experiment with a minimum of 3 microfluidic channels per experiment. Exception is iAstro cAMP with N = 1, n = 5; one independent experiment with 5 microfluidic channels. (F) Relative expression of MMP2 normalized to CD31 as assessed with quantitative real-time PCR (qRT-PCR) for the four VoC culture conditions at end-point day 7. Data shown as mean ± SD from N = 3-6 independent experiments. In the iAstro conditions, data shown is from triple cultures containing both iAstros from the FLB and the TUBA hiPSC line. One-way ANOVA with Sidaks multiple comparison test. *$p < 0.05$ (G) Representative immunofluorescence confocal surface rendered images of microvascular networks in microfluidic chips of VoC cultures on day 7 showing hiPSC-mCherry ECs (red). Scale bars: 100 µm.

SUPPLEMENTAL FIGURE 4.

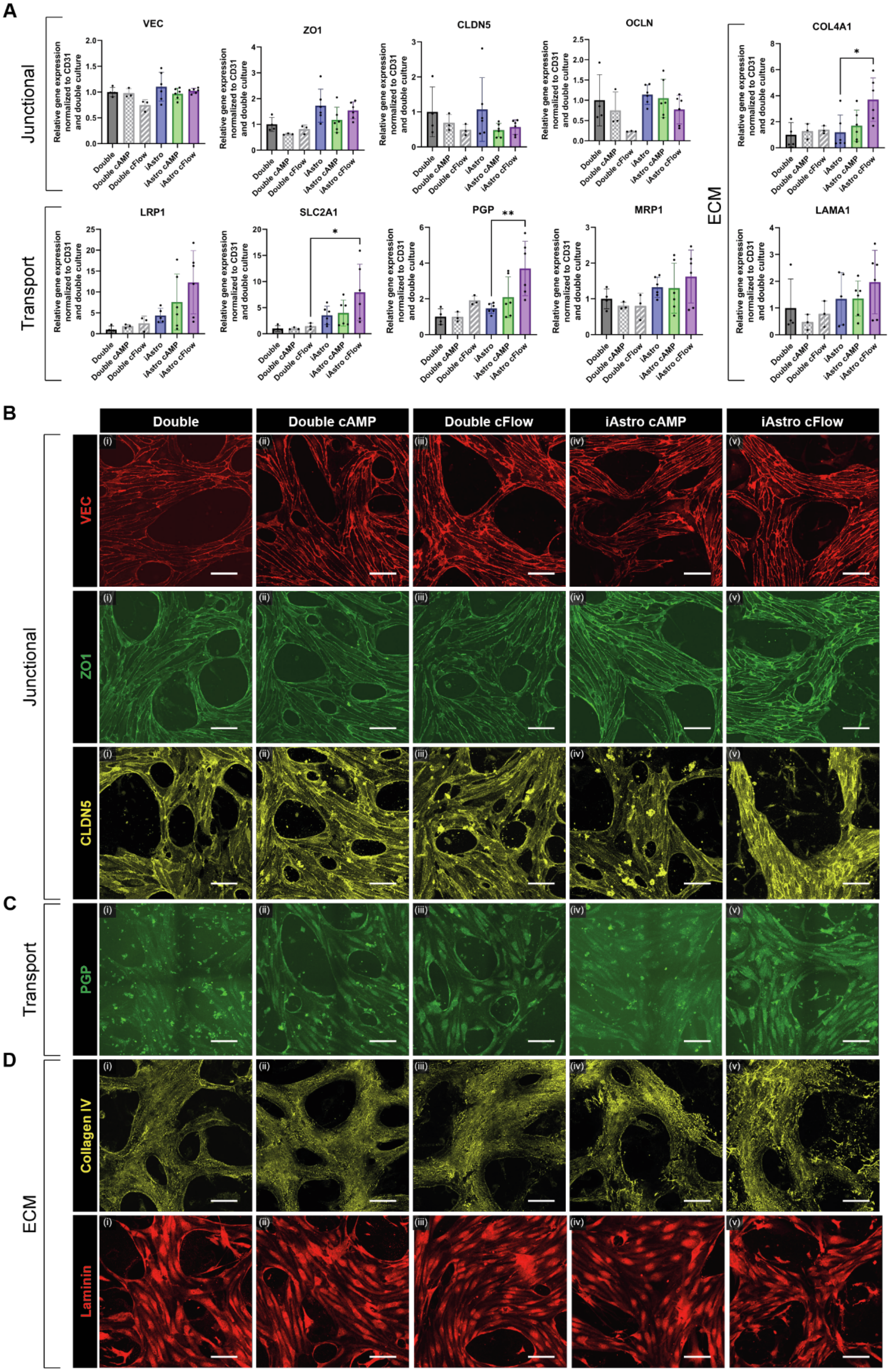

Figure S4. Related to Figure 4. Assessment of blood-brain barrier properties in 3D VoC cultures.
(A) RNA expression of key BBB-genes for the different 3D VoC culture conditions. Values were obtained with qRT-PCR and were normalized to housekeeping gene hARP and to CD31. Individual repeats are plotted for the conditions indicated. (B) Representative immunofluorescence confocal images for junctional proteins VE-Cadherin (VEC), Zonula occludens-1 (ZO1) and Claudin-5 (CLDN5). (C) Representative immunofluorescence confocal images for transporter protein P-glycoprotein (PGP). (D) Representative immunofluorescence confocal images for extracellular matrix (ECM) proteins Collagen IV and Laminin. Scale bars: 100 μm. Data are shown as mean ± SD. For N = 3-4, n = 3-6; three or four independent experiments with one pulled RNA sample per condition. In the iAstro conditions, data is pulled from triple cultures containing both iAstros from the FLB and the TUBA hiPSC lines. One-way ANOVA with Sidaks multiple comparison test. *$p < 0.05$, **$p < 0.01$; ns, non-significant.

Supplemental Table 1. List of hiPSC lines and batches used per experiment

Figure	hiPSC-ECs		hiPSC-Astros	
	Line	Number of batches	Line	Number of batches
Fig S1 F	-	-	iAstro: LUMC0020iCTRL	3
			iAstro: LUMCi005-A	1
			iSCT-Astro LUMC0020iCTRL	2
			iSCT-Astro LUMC114iCTRL01	2
Fig S1 H, I	-	-	LUMC0020iCTRL	3
Fig 1 D-F; 2 B, D, E	NCRM-1	3	LUMC0020iCTRL	2
			AICS-0012	1
Fig 3 C-F	NCRM-1	3	AICS-0012	1
Fig 4 D-F; S3 B-D	NCRM-1	3	LUMC0020iCTRL	2
Fig 4 G, I; S4 A	LUMC0054iCTRL02	1	LUMC0020iCTRL	1
			AICS-0012	1
Fig S4 C, E, G	LUMC0054iCTRL02	1	LUMC0020iCTRL	1

Supplemental Table 2. List of antibodies for IF

Antibody	Species	Source	Use	Dilution	Catalog #
SOX2	Rat	eBiosciences	2D	1:200	53-9811-80
PAX6	Rabbit	Cell Signaling	2D	1:200	60433S
FOXG1	Rabbit	Abcam	2D	1:200	ab18259
GFAP	Rabbit	DAKO	2D + 3D VoC	1:1500	Z033401
FABP7	Mouse	Santa Cruz	2D + 3D VoC	1:500	sc-374588
S100ß	Mouse	Sigma	2D	1:500	S2532
VIM	Mouse	Sigma	2D	1:300	V6630
SLC1A3/GLAST/EAAT1	Mouse	Miltenyi	2D	1:100	130-095-822
Aqp4	Rabbit	Novus Bio	3D VoC	1:200	NBP1-87679
NG2	Mouse	Santa Cruz	3D VoC	1:200	sc-53389
SM22/TAGLN	Rabbit	Abcam	3D VoC	1:400	ab14106
SOX17	Goat	R&D systems	3D VoC	1:300	AF1924
PGP	Mouse	Invitrogen	3D VoC	1:100	MA1-26528
Collagen IV	Goat	Millipore	3D VoC	1:200	AB769
Laminin	Rabbit	Sigma	3D VoC	1:100	AB19012

Supplemental Table 3. List of primers for qRT-PCR

Target	Forward primer (5' – 3')	Reverse primer (5' – 3')
VEC	GGCATCATCAAGCCCATGAA	TCATGTATCGGAGGTCGATGGT
CD31	GCATCGTGGTCAACATAACAGAA	GATGGAGCAGGACAGGTTCAG
ZO1	CAACATACAGTGACGCTTCACA	CACTATTGACGTTTCCCCACTC
CLDN5	GCGTGCTCTACCTGTTTTGC	CAGCTCGTACTTCTGCGACA
OCLN	ACAAGCGGTTTTATCCAGAGTC	GTCATCCACAGGCGAAGTTAAT
SLC2A1	AACTCTTCAGCCAGGGTCCAC	CACAGTGAAGATGATGAAGAC
PGP	TGACCCGCACTTCAGCTAC	GGGCTTCCCGATGATGTCG
MRP1	TTACTCATTCAGCTCGTCTTGTC	CAGGGATTAGGGTCGTGGAT
LRP1	CTATCGACGCCCCTAAGACTT	CATCGCTGGGCCTTACTCT
COL4A1	CAAAAGGGTGATACTGGAGAACC	ATTTCCTGCGAAACCAGGCA
LAMA1	GTGATGGCAACAGCGCAAA	GACCCAGTGATATTCTCTCCCA
MMP2	CTACGATGGAGGCGCTAATGG	CTTGGGGCAGCCATAGAAGG

Supplemental Experimental Procedures

hiPSC lines and maintenance

hiPSCs were maintained on recombinant vitronectin-coated plates in TeSR-E8, all from StemCell Technologies, according to the manufacturer's instructions. hiPSCs used for astrocyte differentiation were cultured on matrigel-coated (BD Biosciences, 354230) plates in TeSR^{TM}1 medium (StemCell Technologies, 05850) and mechanically passaged once a week using dispase solution 1 mg/mL (Gibco, 17105-041). The following hiPSC lines were used: LUMC0020iCTRL (Described in this report as FLB and generated from skin fibroblasts, https://hpscreg.eu/cell-line/LUMCi028-A) (Zhang et al., 2014). NIH Center for Regenerative Medicine hiPSC line (NCRM-1, generated from CD34+ cord blood cells, https://hpscreg.eu/cell-line/CRMi003-A), obtained from RUDCR Infinite Biologics at Rutgers University, was modified in-house with a mCherry expression cassette under the human cytomegalovirus (hCMV) early enhancer/chicken β actin (CAG) promoter using a previously established protocol (Rostovskaya et al., 2012). The Allen Cell Collection line AICS-0012 (Described in this report as TUBA and generated from skin fibroblasts, https://hpscreg.eu/cell-line/UCSFi001-A-2) with mEGFP insertion site at TUBA1B.

Differentiation of hiPSCs towards ECs

hiPSCs were maintained in mTeSR-E8 and differentiated towards ECs as previously described (Orlova et al., 2014b, 2014a). Briefly, mesoderm was induced by changing the media to B(P)EL medium supplemented with 8 μM CHIR99021 (Tocris Bioscience, 4423). Cells were refreshed at day 3, 6 and 9 with B(P)EL with VEGF (50 ng/mL) and 10 μM SB431542 (Tocris Bioscience, 1614). hiPSC-ECs were isolated on day 10 using CD31-Dynabeads™ (Thermo Fisher Scientific) as previously described (Orlova et al., 2014b, 2014a). hiPSC-ECs were expanded in complete EC growth medium comprised of Human Endothelial-serum free medium (EC-SFM) with 1% Human platelet poor serum (P2918, Sigma), VEGF (30 ng/mL) and bFGF (20 ng/mL). hiPSC-ECs were expanded for additional 3-4 days post-isolation and cryopreserved using serum-free cryopreservation medium at passage number 1 (P1) (CryoStor®CS10) (StemCell Technologies, 100-1061).

Differentiation of hiPSCs towards neural progenitors

Neural progenitor cells (NPCs) were generated through a regionalized neural organoid phase as described previously, with minor modifications (Peteri et al., 2021). Briefly, hiPSCs were dissociated using 0,5 mM Ethylenediaminetetraacetic acid (EDTA, Invitrogen, 15575020) and plated as small clumps 1:1 into ultra-

low attachment 6-well plate (Corning, 3471) or ultra-low T75 (Corning, 3814) in mTeSR-1 with 20 ng/mL bFGF (Miltenyi Biotec, 130-093-842) and RevitaCell (Life Technologies, 1:200). Following day an additional refreshment with mTESR-1, bFGF and Revitacell. The subsequent day neural induction and regional (forebrain) patterning was started by changing the medium to Neuronal Induction Medium (NIM) consisting of advanced DMEM/F12 (Life Technologies, 31331028), 2 mM L-glutamine (Life Technologies, 25030), 1% non-essential amino acids (NEAA; Life Technologies, 11140035), 1% N2 supplement (Life Technologies, 17502048), 1% Penicillin-Streptomycin (Life Technologies, 15070063) supplemented with 0.1 µM LDN-193189 (Axon Medchem), 10 µM SB-431542 (Tocris Bioscience, 1614), 0.5 µg/mL DKK-1 (PrepoTech, 120-30B) and 1 µM cyclopamine (R&D systems, 1623/1). Patterning took place from day 0 until day 12 with medium changes every second day. NPCs were matured from day 12 to day 30 by refreshment every second day with NIM medium supplemented with 20 ng/mL brain-derived neurotrophic factor (BDNF; Peprotech, 450-02). NPCs were expanded from day 30 by switching to Neurosphere (NS) Medium consisting of advanced DMEM/F12, 2 mM L-glutamine, 1% NEAA, 2% B27 supplement (Life Technologies, 17504044), 2 µg/mL heparin (Leo Pharma BV, 14179857) and 1% Penicillin-Streptomycin supplemented with 20 ng/mL bFGF and 20 ng/mL epidermal growth factor (EGF; R&D systems, 236-EG-200). Medium was changed two times a week and NPC spheres were manually dissociated to small clumps approximately every one and a half week. At day 60, NPC spheres were manually dissociated to small clumps and cryopreserved in 50 % NS medium, 40 % FBS (Biowest, S1860) and 10 % dimethyl sulfoxide (DMSO; Sigma, D2650). NPCs were thawed and cultured on 6-well plates coated with 20 µg/mL poly-ornithine (PO; Sigma, P3655) and 5 µg/mL laminin (Sigma, L2020) and maintained for 5 days in NS medium supplemented with 20 ng/mL bFGF and 20 ng/mL EGF to recover. The first day the media was also supplemented with 1:200 Revitacell. The NPCs were subsequently used for differentiation towards astrocytes.

Differentiation of neural progenitors towards astrocytes

NPCs were either differentiated using a previously published protocol (Peteri et al., 2021) (iAstros) or using a commercially available kit (iSCT Astros). In the iAstro differentiation, astrocyte specification was started by changing the media to NS medium supplemented with 20 ng/mL CNTF (Peprotech, 450-13). When reaching 80-90% confluency, cultures were passaged 1:4 using accutase (Millipore, SCR005). At day 30, astrocytes were cryopreserved in CryoStor®CS10. For characterization and functional assays of astrocytes, cryopreserved iAstros were thawed and cultured for 3 days on PO/laminin-coated plates in NS medium supplemented with 20 ng/mL CNTF.

For iSCT Astro differentiation, NPCs were passaged to matrigel-coated plates and maintained in STEMdiff™ Neural Progenitor Medium (StemCell Technologies, 05833) for 4-5 days to recover and expand. NPCs were subsequently differentiated using the STEMdiff™ Forebrain Neuron Differentiation Kit (StemCell Technologies, 08600), following manufacturer's protocol. After completing the 7 days differentiation protocol, medium was changed to BrainPhys™ Neuronal medium (StemCell Technologies, 05790) and cells were maintained for 11 more days before using in functional assays. Population of astrocytes was confirmed by positive staining for key astrocyte markers. Cells were passaged using accutase before use in microfluidic chips to enrich the astrocyte population.

Primary human brain vascular pericyte and primary astrocyte culture

Human brain vascular pericytes (HBVPs) and primary human cortical astrocytes (pAstros) were purchased from ScienceCell. HBVPs were cultured in Pericyte Medium (ScienceCell, 1201) supplemented with 1% Pericyte Growth Supplement (ScienceCell, 1252), 2% FBS and 1% penicillin/streptomycin. pAstros were cultured on poly-L-lysin coated (15 µg/mL, Sigma, P4707) plates in Astrocyte Medium (ScienceCell, 1801) supplemented with 1% Astrocyte Growth Supplement (AGS, Sciencell, 1852), 2% FBS and 1% penicillin/streptomycin. HBVPs and pAstros were cryopreserved at passage number 3 (P3) or 2 (P2) respectively, using serum-free cryopreservation medium (CryoStor®CS10) (StemCell Technologies, 100-1061).

Cell preparation prior to VoC culture

hiPSC-ECs (P1) were thawed and cultured on gelatin-coated plates in complete EC growth medium composed of Human Endothelial-SFM (EC-SFM) with 1% platelet poor serum (PPS), VEGF (30 ng/mL) and bFGF (20 ng/mL) 4 days prior to VoC seeding. HBVPs (P4) were thawed and cultured on gelatin-coated plates in Pericyte Medium (ScienceCell, 1201) supplemented with 1% Pericyte Growth Supplement

(ScienceCell, 1252), 2% FBS and 1% penicillin/streptomycin, 4 days prior to VoC seeding. pAstros were thawed and cultured in Astrocyte Medium (ScienceCell, 1801) supplemented with 1% Astrocyte Growth Supplement (AGS, Sciencell, 1852), 2% FBS and 1% penicillin/streptomycin, 4 days prior to VoC seeding. iAstros were thawed and cultured in NS medium supplemented with 20 ng/mL CNTF, 4 days prior to VoC seeding. iSCT-Astros were used directly after maturation at the end of the differentiation protocol. For an overview of the hiPSC lines and differentiation batches used for the different experiments, see Supplemental Table 1.

Immunostaining and microscopy of forebrain neural organoid

Forebrain neural organoids were fixed with 4% paraformaldehyde (PFA, Sigma) for 30 minutes at 4 °C and washed with phosphate-buffered saline (PBS) before continuing for wholemount staining. Cell plasma membranes were permeabilized with 0.5% Triton X-100 for 15 minutes at RT and washed 3 times for 10 minutes with PBS. Blocking was performed by adding 2% BSA in PBS for 3 hours at RT. Primary antibodies (see Supplemental Table 1 for details) were diluted in 1% BSA in PBS and incubated O/N at 4 °C. After washing with PBS, secondary antibodies (1:300, Invitrogen) diluted in 1% BSA were added and incubated for 2 hours at RT. Stained organoids were mounted with ProLong Gold Antifade Mountant (Thermofisher Scientific #P36930) on microscope slides. Images were taken using the EVOS M7000 using 20x magnification objective.

Immunostaining, microscopy and quantification of 2D astrocytes

iAstros were seeded on PO/laminin coated 96-well black imaging plates (Corning) at a seeding density of 35000 cells/well in NS medium supplemented with 20 ng/mL CNTF. iSCT-Astros were seeded on PO/laminin coated 96-well black imaging plates at a seeding density of 35000 cells/well in BrainPhys™ Neuronal medium. Both were fixed 3 days later, using 4% PFA for 10 minutes at RT. Cell membranes were permeabilized with 0.1% Triton X-100 for 5 minutes at RT and washed with PBS before blocking with 1% BSA in PBS for 1 hour. Primary antibody diluted in 1% BSA were added and incubated O/N at 4 °C. For primary antibody overview see Supplemental table 1. Images were taken using EVOS M7000 using 10x magnification objective. Quantification of the percentage of marker positive astrocytes was performed using custom pipelines developed on the free open source CellProfiler software (https://cellprofiler.org/) (Carpenter et al., 2006). In brief, both nuclei and marker objects were identified after pre-processing steps to reduce unspecific object identification. Nuclei and marker objects were subsequently overlapped for visual examination of proper identification and to calculate number of masked nuclei objects.

Immunostaining and microscopy of VoC

Cells in VoCs were fixed *in situ* in 4% PFA for 30 minutes at RT. Cell plasma membranes were permeabilized with 0.5% Triton X-100 for 15 minutes at RT and washed 3 times for 10 minutes with PBS. Blocking was performed by adding 2% BSA in PBS for 3 hours at RT. Primary antibodies (see Supplemental Table 1 for details) were diluted in 1% BSA in PBS and incubated O/N at 4 °C. After washing with PBS, secondary antibodies (1:300, Invitrogen) diluted in 1% BSA were added and incubated for 2 hours at RT. Images of the full microfluidic channel of VoCs were taken with the EVOS M7000 using the 10x objective and automated stitching. 3D images were taken using the DragonFly spinning disk (Andor) microscope with 40x magnification objective, 2x2 tile scans with automated stitching and post-processing performed using Imaris 9.5 software (Bitplane, Oxford Instruments).

EdU assay for EC proliferation in 3D microfluidic chips

Proliferation was measured using the EdU Click-iT kit Alexa-488 (ThermoFisher Scientific #C10337) according to manufacturer's protocol. Briefly, on day 4 of culture, microfluidic chips were refreshed with EGM-2 supplemented with 50 ng/mL VEGF and 1% AGS additionally supplemented with EdU (1:1000) for 6 hours. Cells were fixed with 4% PFA for 30 minutes, permeabilized with 0.5% TX-100 for 15 minutes at RT. Freshly prepared Click-iT reaction cocktail was added for 3 hours at RT. Microfluidic chips were washed three times with PBS and blocked in 2% BSA in PBS for 3 hours at RT, followed by co-staining with primary and secondary antibodies.

Characterization of vascular and perivascular parameters in 2D images

Quantification of VoC vascular and perivascular parameters vessel density, average diameter, average vessel length, average astrocyte length and number, average HBVP length and number, EdU+ nuclei,

EdU+ ECs of total EdU+ nuclei, EdU+ ECs, PGP intensity and CollagenIV and Laminin area from 2D images was performed as previously described (Orlova et al., 2022; Vila Cuenca et al., 2021). Briefly, images of the whole microfluidic channel as acquired using EVOS M7000, were quantified using custom pipelines developed on the free open source CellProfiler software (https://cellprofiler.org/) (Carpenter et al., 2006). Pre-processing steps were applied to all images to enhance image features and a gaussian filter to reduce unspecific object identification. A minimum cross-entropy thresholding method was used on vascular network images to produce a binarized image. The binarized images from the CellProfiler output were then analyzed using ImageJ software with the freely available plugin DiameterJ (https://imagej.nih.gov/ij/, https://imagej.net/DiameterJ) (Hotaling et al., 2015). For quantification of CollagenIV and Laminin, a similar pipeline was used to generate binarized images. Quantification of PGP intensity was done by using a custom cell profiler pipeline with maximum projection images from 3D confocal images as input. Both intensity of PGP and area covered by either CollagenIV or Laminin staining was normalized by the area of the vessel, as determined by CD31 staining . EdU+ nuclei, EdU+ ECs of total EdU+ nuclei and EdU+ ECs were quantified with a custom-made pipeline in CellProfiler (Carpenter et al., 2006).

Characterization of astrocyte and HBVP cell parameters in 3D

Characterization and quantification of GFAP positive and SM22 positive objects and quantification of PGP intensity was performed by 3D quantitative analysis using images taken with the DragonFly spinning disk (Andor) and processed using Imaris 9.5 software (Bitplane, Oxford Instruments). Average HBVP SM22 object intensity and volume was obtained by first surface-rendering both individual SM22 positive objects and individual FABP7 or TUBA-GFP positive objects for the pAstro or iAstro conditions respectively. HBVP SM22 positive objects were obtained by filtering out the double positive objects. For quantification of the percentage of HBVP SM22 positive or GFAP positive objects touching the vessel, first an additional surface rendering of the microvascular network (mCherry positive) was performed. HBVP SM22 positive or GFAP positive objects touching the vessel was defined as a distance of 0 µm between the surface-rendered objects and >0 µm distance was defined as no contact.

Perfusion assessment in VoC system

Before the perfusion assessment and time-lapse imaging for permeability quantification, ECs were first stained using Ulex Europaeus Agglutinin I, DyLight594 (1:600, Vector Laboratories, DL-1067) by incubation of the microfluidic channels for 45 minutes in the incubator. Subsequently, time-lapse images were taken using the EVOS M7000 with on stage incubator with the 10x objective at 20 fps for 30 seconds. 70 µL of 70 KDa FITC-Dextran (1:1000, Sigma) in EGM-2 was added to one medium port and 50 µL of EGM- 2 to all other media ports to induce interstitial gravity driven flow. Agglutinin was imaged at the same location as the dextran perfusion was imaged to enable accurate assessment of fluorescent tracer leakage inside and outside the microvascular network. Calculation of permeability coefficient was based on previously established methods (Hajal et al., 2022). In short, the following formula was used:

$$P = \frac{1}{\Delta t}\frac{Am}{SPv}\frac{\Delta Im}{\Delta I}$$

With $\Delta t = T2 - T1$ (30 seconds), *Am* being the surface area of the matrix, *SPv* being the surface perimeter of the vessel, $\Delta Im = (Im2 * (Iv1/Iv2)) - Im1$ being the difference in fluorescence intensity in the matrix corrected for potential changes in the dextran concentration in the vascular space during imaging and $\Delta I = Iv1 - Im1$ being the difference in fluorescence intensity between the vasculature and matrix at the start of the measurement.

Assessment of intracellular Ca2+ release in astrocytes

Intracellular Ca^{2+} release was assessed in astrocytes at day one post-seeding in a black, flat-bottomed 96-well plate coated with poly-l-lysine for pAstros and PO/laminin for iAstros. The calcium-6 dye (Molecular Devices) was dissolved in 10 mL HBSS buffer B and subsequently diluted 1:4 in Buffer B (Molecular Devices). The diluted dye solution was added 1:1 to the wells containing astrocytes in NS medium. Astrocytes were incubated for 2 hours at 37 °C with 5% CO_2 before being measured on the FDSS/µcell (Hamamatsu Photonics) at 37 °C with an exposure time of 0.017s. Response to ATP stimulus was performed by first preparing a “compound plate” including a medium control of NS medium, 30 µM ATP (Sigma, A9187) in NS medium or 3 mM ATP in NS medium. Control and ATP stimulus from the “compound

plate" were automatically mixed and injected into the assay plate, reaching final ATP concentrations of 3 and 300 µM. Analysis was performed in R (4.0.3) and the induced change in Ca^{2+} release was calculated by quantification of the area under the curve of the average fluorescence intensity normalized to the NS medium control.

Glutamate uptake assay
A colorimetric glutamate assay kit (Sigma, MAK004) was used to determine the reduction of glutamate in the cell culture medium over time. Cells were plated 2 days before the assay in a 96 well plate. Before the assay, cells were washed with HBSS (Gibco) and then incubated with 100 µM glutamate in HBSS. Samples were collected and analyzed according to the manufacturer's instructions. The uptake of glutamate was normalized to the number of cells per well. Cells were stained with 1 µg/mL HOECHST 33342 (Thermo Fisher Scientific, 62249) for 20 minutes before washing with PBS. Whole wells were imaged using EVOS M7000 and subsequent downstream identification and quantification of the number of nuclei was performed with ImageJ software (https://imagej.nih.gov/ij/).

RNA isolation and quantitative RT-PCR
Total RNA was isolated from the microfluidic devices at end-point day 7. Cells were extracted by dissolving the extracellular matrix / fibrin mix with Collagenase B (1 mg/ml, Roche, 11088815001) for half an hour at 37 degrees °C, while rocking. RNA was extracted using the NucleoSpin RNA XS kit (Macherey-Nagel) and cDNA was synthesized using an iScript-cDNA Synthesis kit (Bio-Rad). iTaq Universal SYBR Green Supermixes (Bio-Rad) and Bio-Rad CFX384 real-time system were used for the PCR reaction and detection. Primers used can be found in Supplemental Table 3. Relative gene expression was calculated using the delta Ct calculation and normalized to the housekeeping gene hARP and to CD31. Heatmap was generated using the freely available online tool http://www.heatmapper.ca/expression/ (Babicki et al., 2016).

Supplemental References

Babicki, S., Arndt, D., Marcu, A., Liang, Y., Grant, J.R., Maciejewski, A., and Wishart, D.S. (2016). Heatmapper: web-enabled heat mapping for all. Nucleic Acids Res *44*, W147–W153. https://doi.org/10.1093/NAR/GKW419.

Carpenter, A.E., Jones, T.R., Lamprecht, M.R., Clarke, C., Kang, I.H., Friman, O., Guertin, D.A., Chang, J.H., Lindquist, R.A., Moffat, J., et al. (2006). CellProfiler: Image analysis software for identifying and quantifying cell phenotypes. Genome Biol *7*. https://doi.org/10.1186/gb-2006-7-10-r100.

Hajal, C., Offeddu, G.S., Shin, Y., Zhang, S., Morozova, O., Hickman, D., Knutson, C.G., and Kamm, R.D. (2022). Engineered human blood–brain barrier microfluidic model for vascular permeability analyses. Nat Protoc *17*, 95–128. https://doi.org/10.1038/s41596-021-00635-w.

Hotaling, N.A., Bharti, K., Kriel, H., and Simon, C.G. (2015). DiameterJ: A validated open source nanofiber diameter measurement tool. Biomaterials *61*, 327–338. https://doi.org/10.1016/j.biomaterials.2015.05.015.

Orlova, V. V., van den Hil, F.E., Petrus-Reurer, S., Drabsch, Y., ten Dijke, P., and Mummery, C.L. (2014a). Generation, expansion and functional analysis of endothelial cells and pericytes derived from human pluripotent stem cells. Nat Protoc *9*, 1514–1531. https://doi.org/10.1038/nprot.2014.102.

Orlova, V. V., Drabsch, Y., Freund, C., Petrus-Reurer, S., Van Den Hil, F.E., Muenthaisong, S., Ten Dijke, P., and Mummery, C.L. (2014b). Functionality of endothelial cells and pericytes from human pluripotent stem cells demonstrated in cultured vascular plexus and zebrafish xenografts. Arterioscler Thromb Vasc Biol *34*, 177–186. https://doi.org/10.1161/ATVBAHA.113.302598.

Orlova, V. V., Nahon, D.M., Cochrane, A., Cao, X., Freund, C., van den Hil, F., Westermann, C.J.J., Snijder, R.J., Ploos van Amstel, J.K., ten Dijke, P., et al. (2022). Vascular defects associated with

hereditary hemorrhagic telangiectasia revealed in patient-derived isogenic iPSCs in 3D vessels on chip. Stem Cell Reports *17*, 1536–1545. https://doi.org/10.1016/j.stemcr.2022.05.022.

Peteri, U., Pitkonen, J., Utami, K.H., Paavola, J., Roybon, L., Pouladi, M.A., and Castren, M.L. (2021). Generation of the Human Pluripotent Stem-Cell-Derived Astrocyte Model with Forebrain Identity. Brain Sci *11*.

Rostovskaya, M., Fu, J., Obst, M., Baer, I., Weidlich, S., Wang, H., Smith, A.J.H., Anastassiadis, K., and Francis Stewart, A. (2012). Transposon-mediated BAC transgenesis in human ES cells. Nucleic Acids Res *40*. https://doi.org/10.1093/nar/gks643.

Vila Cuenca, M., Cochrane, A., van den Hil, F.E., de Vries, A.A.F., Lesnik Oberstein, S.A.J., Mummery, C.L., and Orlova, V. V. (2021). Engineered 3D vessel-on-chip using hiPSC-derived endothelial- and vascular smooth muscle cells. Stem Cell Reports *16*. https://doi.org/10.1016/j.stemcr.2021.08.003.

Zhang, M., D'Aniello, C., Verkerk, A.O., Wrobel, E., Frank, S., Ward-Van Oostwaard, D., Piccini, I., Freund, C., Rao, J., Seebohm, G., et al. (2014). Recessive cardiac phenotypes in induced pluripotent stem cell models of Jervell and Lange-Nielsen syndrome: Disease mechanisms and pharmacological rescue. Proc Natl Acad Sci U S A *111*, E5383–E5392. https://doi.org/10.1073/pnas.1419553111.
