# Supplementary material for: Self-assembling 3D vessel-on-chip model with hiPSC-derived astrocytes
Source: Stem Cell Reports. 2024 Jun 13;19(7):946–56. doi: 10.1016/j.stemcr.2024.05.006 (PMC11252484; doi:10.1016/j.stemcr.2024.05.006)
Supplement: Document S2. Article plus supplemental information [file mmc3.pdf]

Stem Cell Reports

Report

OPEN ACCESS

Self-assembling 3D vessel-on-chip model with hiPSC-derived astrocytes

Dennis M. Nahon,[1] Marc Vila Cuenca,[1,2] Francijna E. van den Hil,[1] Michel Hu,[3,4] Tessa de Korte,[1] Jean-Philippe Frimat,[3,4] Arn M.J.M. van den Maagdenberg,[3,4] Christine L. Mummery,[1] and Valeria V. Orlova[1,5,*]

[1]Department of Anatomy and Embryology, Leiden University Medical Centre, 2333ZA Leiden, the Netherlands
[2]Department of Clinical Genetics, Leiden University Medical Centre, 2333ZA Leiden, the Netherlands
[3]Department of Human Genetics, Leiden University Medical Centre, 2333ZA Leiden, the Netherlands
[4]Department of Neurology, Leiden University Medical Centre, 2333ZA Leiden, the Netherlands
[5]Lead contact
*Correspondence: v.orlova@lumc.nl
https://doi.org/10.1016/j.stemcr.2024.05.006

SUMMARY

Functionality of the blood-brain barrier (BBB) relies on the interaction between endothelial cells (ECs), pericytes, and astrocytes to regulate molecule transport within the central nervous system. Most experimental models for the BBB rely on freshly isolated primary brain cells. Here, we explored human induced pluripotent stem cells (hiPSCs) as a cellular source for astrocytes in a 3D vessel-on-chip (VoC) model. Self-organized microvascular networks were formed by combining hiPSC-derived ECs, human brain vascular pericytes, and hiPSC-derived astrocytes within a fibrin hydrogel. The hiPSC-ECs and pericytes showed close interactions, but, somewhat unexpectedly, addition of astrocytes disrupted microvascular network formation. However, continuous fluid perfusion or activation of cyclic AMP (cAMP) signaling rescued the vascular organization and decreased vascular permeability. Nevertheless, astrocytes did not affect the expression of proteins related to junction formation, transport, or extracellular matrix, indicating that, despite other claims, hiPSC-derived ECs do not entirely acquire a BBB-like identity in the 3D VoC model.

INTRODUCTION

The blood-brain barrier (BBB) is formed through direct interactions between endothelial cells (ECs), pericytes, and astrocytes in the central nervous system. BBB dysfunction is increasingly recognized as a contributor to multiple neurodegenerative diseases (Sweeney et al., 2018). This has led to many attempts to develop human *in vitro* models that recapitulate complex interactions between astrocytes and the vasculature (Hajal et al., 2021). Some aspects of the BBB *in vitro*, such as high transendothelial electrical resistance (TEER) and low permeability to soluble tracers, have been achieved by co-culturing primary brain microvascular- or cord blood-derived ECs with brain pericytes and astrocytes on a porous membrane (Boyer-Di Ponio et al., 2014; Cecchelli et al., 2014). However, primary brain cells are difficult to obtain and, even from commercial sources, show batch-to-batch variability. Human induced pluripotent stem cell (hiPSC)-derived brain microvascular ECs (BMECs) (Lippmann et al., 2012) have been widely used in engineering approaches for the BBB (Hajal et al., 2021). However, it later turned out that these actually resembled epithelial cells rather than ECs (Lu et al., 2021); this explained their abnormally high TEER values. More recently, alternative protocols to differentiate brain-like microvascular ECs that more closely resemble true ECs based on the expression of EC-specific markers and responses to proinflammatory stimuli have been developed (Gastfriend et al., 2021; Nishihara et al., 2020). These were useful for studying intrinsic defects in hiPSC-ECs derived from multiple sclerosis (MS) patient (Nishihara et al., 2022). In addition, several vessel-on-chip (VoC) models have been developed which combine the three most important cell types of the BBB: ECs, pericytes, and astrocytes. However, while they have some value, some of these models lack the direct heterotypic cell-cell and cell-matrix interactions typically seen *in vivo* (Maoz et al., 2018; Vatine et al., 2019). The models that do recapitulate these interactions using vasculogenesis or angiogenesis as starting points usually use primary cells including primary astrocytes (pAstros) (Campisi et al., 2018; Lee et al., 2020; Winkelman et al., 2022).

In this study, we aimed to develop an hiPSC-based 3D VoC model that integrates ECs from hiPSCs (hiPSC-ECs), human brain vascular pericytes (HBVPs), and hiPSC-derived astrocytes (hiPSC-Astros). We used our earlier protocol to differentiate ECs from hiPSCs (Orlova et al., 2014) and showed that astrocytes derived from hiPSCs could be integrated into the VoC and behaved much like human pAstros. hiPSC-Astros incorporated into a VoC triple culture containing both hiPSC-ECs and HBVPs self-assembled into microvascular networks in 3D with hiPSC-Astros and HBVPs assuming positions surrounding the vascular wall in the microfluidic chip. We also investigated two ways of improving microvascular network formation and organization in the VoC model with hiPSC-Astros: activating the cyclic AMP (cAMP) pathway or introducing continuous microfluidic flow. Despite

This is an open access article under the CC BY license (http://creativecommons.org/licenses/by/4.0/).

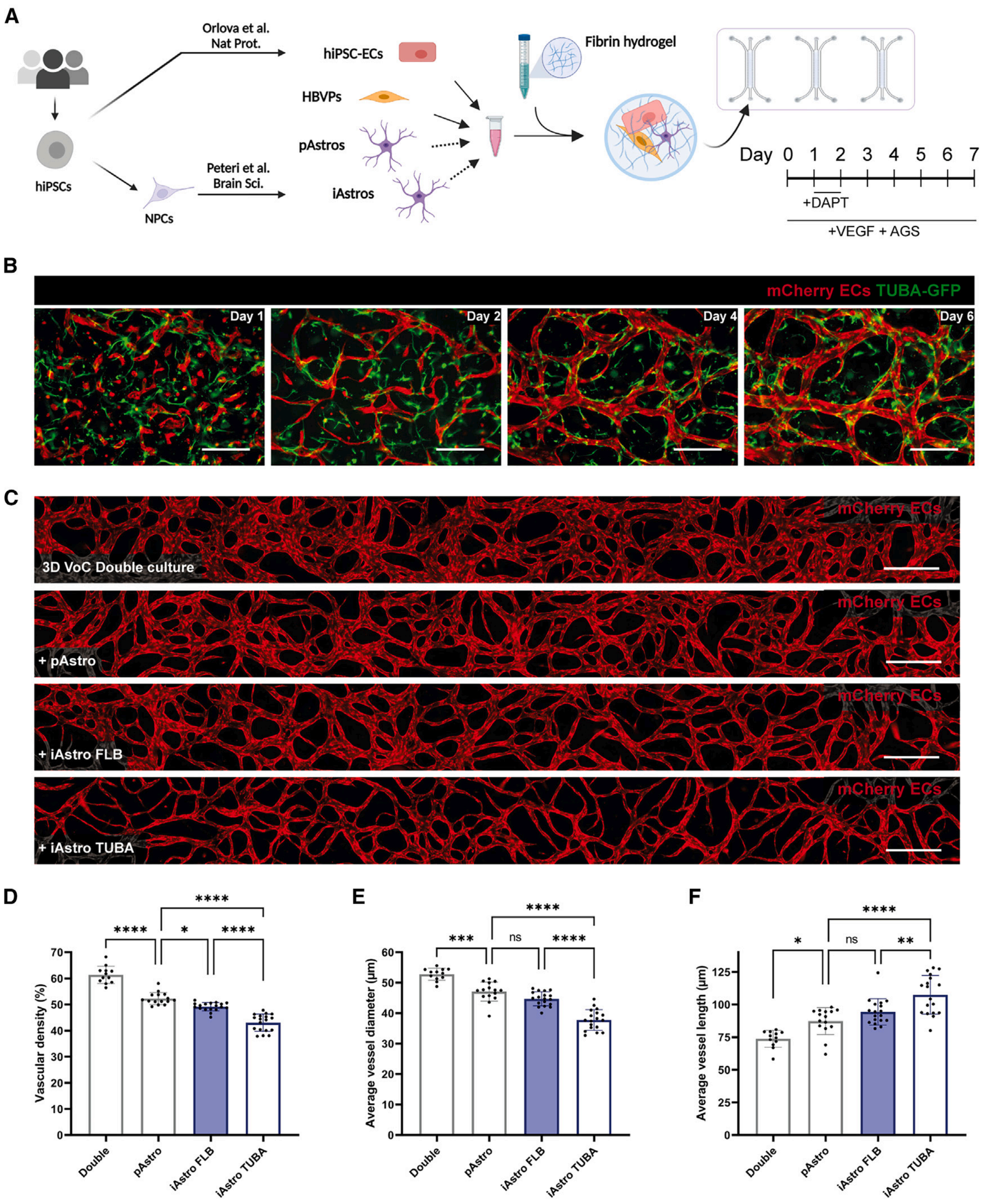

Figure 1. iAstros incorporated into 3D VoC model

(A) Schematic of VoC protocol using hiPSC-ECs with HBVPs and pAstros or iAstros.

(B) Representative immunofluorescence images of a 3D VoC triple culture containing hiPSC-ECs, HBVPs, and iAstros at day 1, 2, 4, and 6 showing hiPSC-mCherry ECs (red) and iAstros differentiated from the TUBA hiPSC line (green, TUBA-GFP). Scale bars: 250 µm.

(C) Representative immunofluorescence images of microvascular networks in microfluidic chips on day 7 showing hiPSC-mCherry ECs (red). Images showing microvascular networks from a VoC double culture (hiPSC-ECs and HBVPs) or 3D VoC triple cultures including either pAstros or iAstros from two independent hiPSC lines (FLB or TUBA). Scale bars: 250 µm.

(legend continued on next page)

improvements in relevant cell interactions and reproducibility, the model still falls short in reproducing a true BBB.

RESULTS

3D VoC integrating hiPSC-Astros

A 3D VoC model was established by combining hiPSC-ECs and HBVPs in a fibrin hydrogel and injecting the cell/gel mix into a commercially available AIM Biotech idenTx9 3D culture chip using a protocol described previously (Vila Cuenca et al., 2021) (Figure 1A). Either hiPSC-Astros or pAstros were included in the VoC model to mimic BBB cell combinations. First, hiPSC-derived neural progenitor cells (NPCs) were generated as previously described (Peteri et al., 2021) (Figure S1A). hiPSC-derived neural organoids contained neural rosette-like structures (Figure S1B), and more of these organoids could be formed in the expansion phase (Figure S1C). Proper patterning and differentiation were confirmed by the expression of the NPC markers SRY-box transcription factor 2 (SOX2) and paired box 6 (PAX6) and the forebrain marker forkhead box G1 (FOXG1) (Figure S1D). We then derived hiPSC-Astros in two different ways: one already published (Peteri et al., 2021) (iAstros) and the other using a commercially available kit (iSCT Astros) (Figure S1A). iAstros and iSCT Astros showed comparable expression of key astrocyte markers, such as glial fibrillary acidic protein (GFAP), fatty acid binding protein 7 (FABP7), S100 calcium binding protein B (S100β), vimentin, and solute carrier family 1 member 3 (SLC1A3/GLAST) (Figures S1E and S1F). In addition, iAstros showed increased intracellular Ca^{2+} release upon stimulation with adenosine triphosphate (ATP) (3 μM and 300 μM) (Figure S1G and S1H) and efficient uptake of the neurotransmitter glutamate (Figure S1F), confirming their functionality.

A triple culture of hiPSC-ECs derived from a control mCherry reporter hiPSC line, HBVPs, and iAstros derived from a control hiPSC line with an α-tubulin-mEGFP reporter (AISC0012, TUBA) (Roberts et al., 2017) was monitored from day 1 to day 6 (Figure 1B). hiPSC-ECs self-organized into interconnected microvascular networks within 2–3 days, with fully lumenized structures by day 7 (Figure 1B). In addition, iAstros localized in the extravascular space, interacting directly with the developing microvascular network (Figure 1B). The development of a robust VoC triple culture model was confirmed by similarly including iSCT Astros from two independent hiPSC lines (FLB or TUBA) (Figure S2). The remaining experiments were performed using iAstros, and not iSCT Astros, as iAstros can be cryopreserved at the endpoint of differentiation and used as a convenient cell source for 3D VoC triple culture setups. The vascular beds thus established were compared between VoC double cultures (only containing hiPSC-ECs and HBVPs) and VoC triple cultures including either pAstros or iAstros from two independent hiPSC lines (FLB or TUBA). All cell combinations formed an interconnected microvascular network by day 7 in a highly reproducible manner across independent experiments (Figures 1C–1F). Quantification of vessel parameters showed that inclusion of any astrocyte source in our VoC model reduced vascular density (%, Figure 1D) and average vessel diameter (μm, Figure 1E) and increased average vessel length (μm, Figure 1F) relative to the double cultures. Significant differences were observed in vessel parameters between pAstro and iAstro triple cultures depending on the hiPSC line used. This is in line with batch-to-batch variability of pAstros previously described (Hajal et al., 2022). Specifically, incorporating iAstros from either the FLB or TUBA hiPSC line into our VoC model resulted in a significant decrease in vascular density compared to pAstros (Figure 1D). However, only iAstros from the TUBA hiPSC line caused a significant decrease in average vessel diameter and increase in average vessel length (Figures 1D–1F).

Characterization of astrocytes and HVBPs in the 3D VoC model

We next examined astrocyte morphology and interaction with hiPSC-EC in microvascular networks in the VoC model. Both pAstros and iAstros from either the FLB or TUBA hiPSC lines stained positively for GFAP and showed uniform distribution through the entire microfluidic channel with no significant differences in the total number of GFAP-positive cells (Figures 2A and 2B). Astrocytes in all conditions showed a distinct stellate morphology and were positioned closely to the abluminal side of the microvascular networks (Figure 2C, Video S1). No significant differences in the average astrocyte length (μm, Figure 2D) or the percentage of astrocytes associated with the microvascular network (%, Figure 2E) were observed between the different sources of astrocytes upon quantification of the confocal images. In addition, iAstros stained positively for the astrocyte-specific water channel aquaporin 4 (AQP4) (Figure 2F), although the staining was distributed across the plasma membrane without polarized expression in the astrocyte endfoot.

(D–F) Quantification of full channel images of microvascular networks showing vascular density at endpoint day 7 showing (D), average vessel diameter (E), and average vessel length (F). Data are shown as mean ± SD. All conditions are $N = 3$, $n = 12–18$; three independent experiments with minimum of 3 microfluidic channels per experiment. One-way ANOVA with Sidak's multiple comparison test. *$p < 0.05$, **$p < 0.01$, ***$p < 0.001$, ****$p < 0.0001$; ns, non-significant. See also Figures S1 and S2.

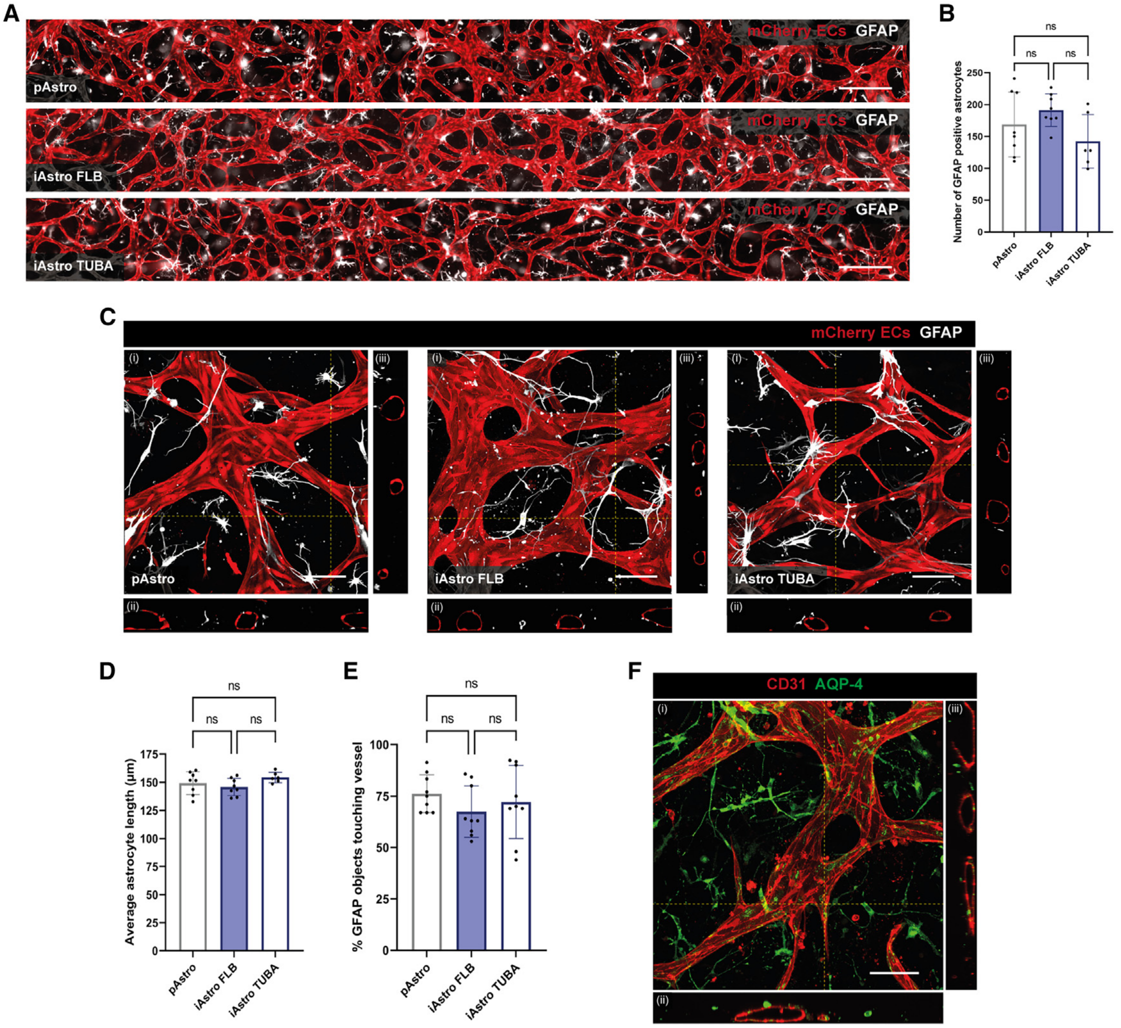

Figure 2. Comparable structural properties of primary and hiPSC-Astros in a 3D VoC model

(A) Representative immunofluorescence images of microvascular networks in microfluidic chips on day 7 showing hiPSC-mCherry ECs (red) and astrocytes (silver; GFAP). Images showing VoC triple cultures of hiPSC-ECs with HBVPs and pAstros or iAstros from FLB or TUBA hiPSC line. Scale bars: 500 μm.

(B) Quantification of astrocytes in VoC triple cultures showing number of GFAP-positive astrocytes in ±80% of full microfluidic channel. ns, non-significant.

(C) Representative immunofluorescence confocal images of microvascular networks in microfluidic chips showing hiPSC-mCherry ECs (red) and astrocytes (silver; GFAP). Images displaying xyz (i), xy (ii), and yz cross-sectional perspectives (iii). Images showing VoC triple cultures of hiPSC-ECs with HBVPs and pAstros or iAstros from the FLB or TUBA hiPSC lines. Scale bars: 100 μm.

(D and E) Quantification of astrocytes in VoC model showing average astrocyte length (D) and % of GFAP objects touching the microvascular network (E). Data are shown as mean ± SD. For (B) and (D) $N = 3$, n = 6–8; three independent experiments with a minimum of two microfluidic channels per experiment. For (E) $N = 3$, $n = 3$; three independent experiments, one microfluidic channel per experiment with three regions of interest (ROIs) per channel. One-way ANOVA with Tukey's multiple comparison. ns, non-significant.

(F) Representative immunofluorescence confocal image of microvascular network in microfluidic chips showing ECs (red; CD31) and astrocytes (green; Aqp4) in a VoC triple culture of hiPSC-ECs with HBVPs and iAstros from the FLB hiPSC line. Images displaying xyz (i), xy (ii), and yz cross-sectional perspectives (iii). Scale bar: 100 μm. See also Video S1.

We confirmed the identity of the HBVPs in the 3D VoC culture by overlaying immunostaining for the pericyte marker neuron-glial antigen 2 (NG2) and contractile marker smooth muscle protein 22 (SM22) (Figure 3A). We previously demonstrated that SM22 is indicative of heterotypic cell-cell contact-induced HBVP cell maturation in VoC cultures (Vila Cuenca et al., 2021) and confirmed similar cell-cell interactions in our current

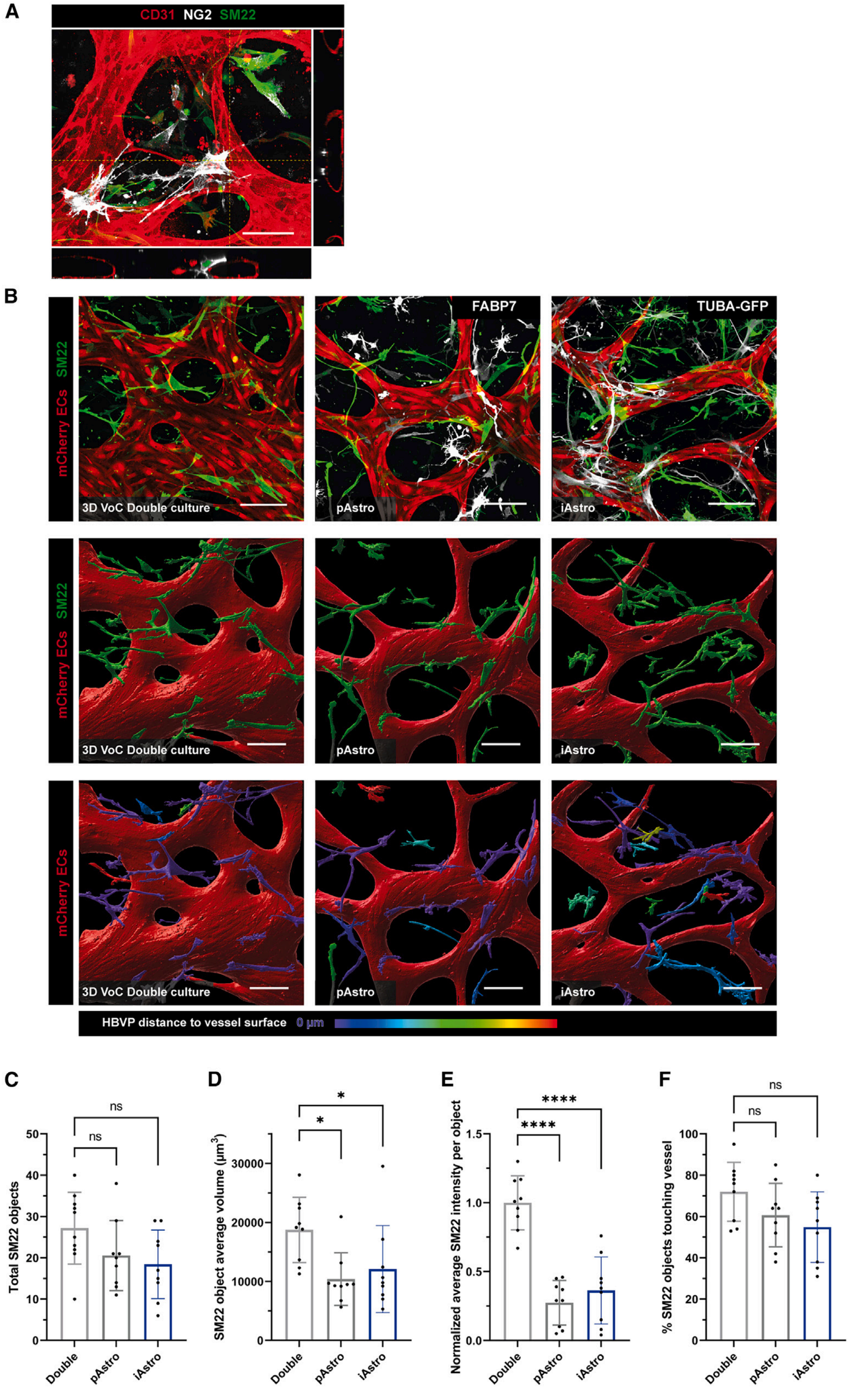

(legend on next page)

VoC setup (Video S1). By using SM22, we therefore investigated whether HBVPs were affected by the addition of astrocytes. Staining of the VoC triple cultures showed SM22 in both HBVPs and astrocytes. To distinguish HBVPs from astrocytes, we used the fluorescently tagged TUBA-GFP iAstros and co-stained the VoC model including pAstros with the glial marker FABP7. Surface rendering of confocal images and identification of SM22+ HBVPs revealed similar numbers of HBVPs in the VoC double cultures and VoC triple cultures including pAstro or iAstros (Figures 3B and 3C). SM22+ HBVPs in the microvascular network had a reduced average volume and normalized average SM22 intensity in the VoC triple cultures with astrocytes (μm^3, Figures 3D and 3E). No significant differences were observed in the direct interaction of the HBVPs with the microvascular network between the different conditions (Figures 3B; %, 3F).

Continuous perfusion or activation of cAMP signaling improves vascular organization and reduces permeability in the 3D VoC model

In addition to reduced microvascular network density and diameter and SM22 volume and intensity, more detailed examination showed local disruptions in the EC layer in both pAstro and iAstro triple culture conditions, although not in the double culture condition (Figure S3A). To improve the triple culture VoC model, we investigated whether culture conditions, postulated to modulate maturation of astrocytes, or microfluidic flow might improve organization of the microvascular networks (Figure 4A). Specifically, we investigated the effect of activation of the cAMP pathway since it has not only been reported to improve astrocyte maturity and immune response (Reuschlein et al., 2019; Zhou et al., 2019) but also shown to be protective for endothelial integrity and barrier function in the BBB (Viña et al., 2021). The influence of continuous flow was studied since mechanical forces resulting from luminal flow through blood vessels are known to promote EC survival, migration, and proliferation (Campinho et al., 2020). VoC triple cultures including iAstros were thus either supplemented daily with dibutytyl cAMP (dbcAMP) (250 μM), the cell membrane-permeable analog of cAMP, or subjected to continuous flow from day 3 till day 7 (Figure 4A). Both dbcAMP addition and continuous flow in VoC triple cultures including iAstros increased vascular density over time (%, Figure 4B). This was most evident on day 7, where both the cAMP and continuous flow conditions significantly increased vascular density and average vessel diameter and decreased average vessel length in comparison to standard VoC triple cultures (Figure 4C; %, 4D; μm, 4E; μm, 4F).

We subsequently investigated whether dbcAMP or continuous perfusion affected microvascular network formation through increased proliferation or through increased presence of matrix metalloproteinase-2 (MMP2), as was shown in a recent study of a VoC containing primary brain ECs, HBVPs, and pAstros (Zhang et al., 2022). Proliferation was quantified by 5-ethynyl-2′-deoxyuridine (EdU) pulse experiments on day 4 of culture. Continuous flow in the VoC model with iAstros increased proliferation compared to the control VoC cultures with or without (dbcAMP-treated) iAstros (Figures S3B and S3C). Co-staining of microfluidic channels with the EC-specific transcription factor SOX17 revealed that most of proliferating cells were hiPSC-ECs (Figures S3D and S3E). Quantitative real-time PCR (real-time qPCR) of VoC cultures at endpoint day 7 confirmed the increase in MMP2 in the iAstro VoC triple culture under continuous flow conditions (Figure S3F).

We next perfused VoC cultures with fluorescein isothiocyanate (FITC)-dextran (70 kDa) to investigate local vascular barrier integrity and quantified the permeability coefficient of the four VoC triple culture conditions (Figures 4G and 4H; cm/s). Both addition of dbcAMP and application of continuous flow improved local vascular integrity and significantly decreased the permeability coefficient in iAstro VoC triple cultures (Figure S3G).

Finally, we explored the influence of the different VoC conditions on the expression of BBB-related markers. Real-time qPCR of VoC culture conditions at endpoint day 7 was somewhat variable between experiments and did not show a significant increase in most of the

Figure 3. Comparable structural properties of HBVPs in a 3D VoC model including astrocytes

(A) Representative immunofluorescence confocal image of microvascular network in microfluidic chips showing ECs (red; CD31) and HBVPs (silver; NG2, green; SM22) in a VoC double culture of hiPSC-ECs with HBVPs. Images displaying xyz (i), xy (ii), and yz cross-sectional perspectives (iii). Scale bar: 100 μm.

(B) Representative immunofluorescence confocal images of microvascular networks in microfluidic chips on day 7 showing hiPSC-mCherry ECs (red), HBVPs (green; SM22), and pAstros (silver, FABP7) or iAstros from the TUBA hiPSC line (silver, TUBA-GFP), surface-rendered images and color-coded images of HBVPs distance to the vessel surface. Images showing VoC double cultures (hiPSC-ECs with HBVPs) and VoC triple cultures including either pAstros or iAstros from the TUBA hiPSC line. Scale bars: 100 μm.

(C–F) Quantification of HBVPs in VoC double and triple cultures containing iAstros from the TUBA hiPSC line showing number of SM22-positive objects (C), average SM22 object volume (D), normalized average SM22 intensity per object (E), and percentage of SM22 objects touching the microvascular network (F). Data are shown as mean ± SD. For (C–F) $N = 3$, $n = 9–10$; three independent experiments, one microfluidic channel per experiment with three or four ROIs per channel. Scale bars: 100 μm. One-way ANOVA with Sidak's multiple comparison test. $*p < 0.05$, $****p < 0.0001$; ns, non-significant. See also Video S1.

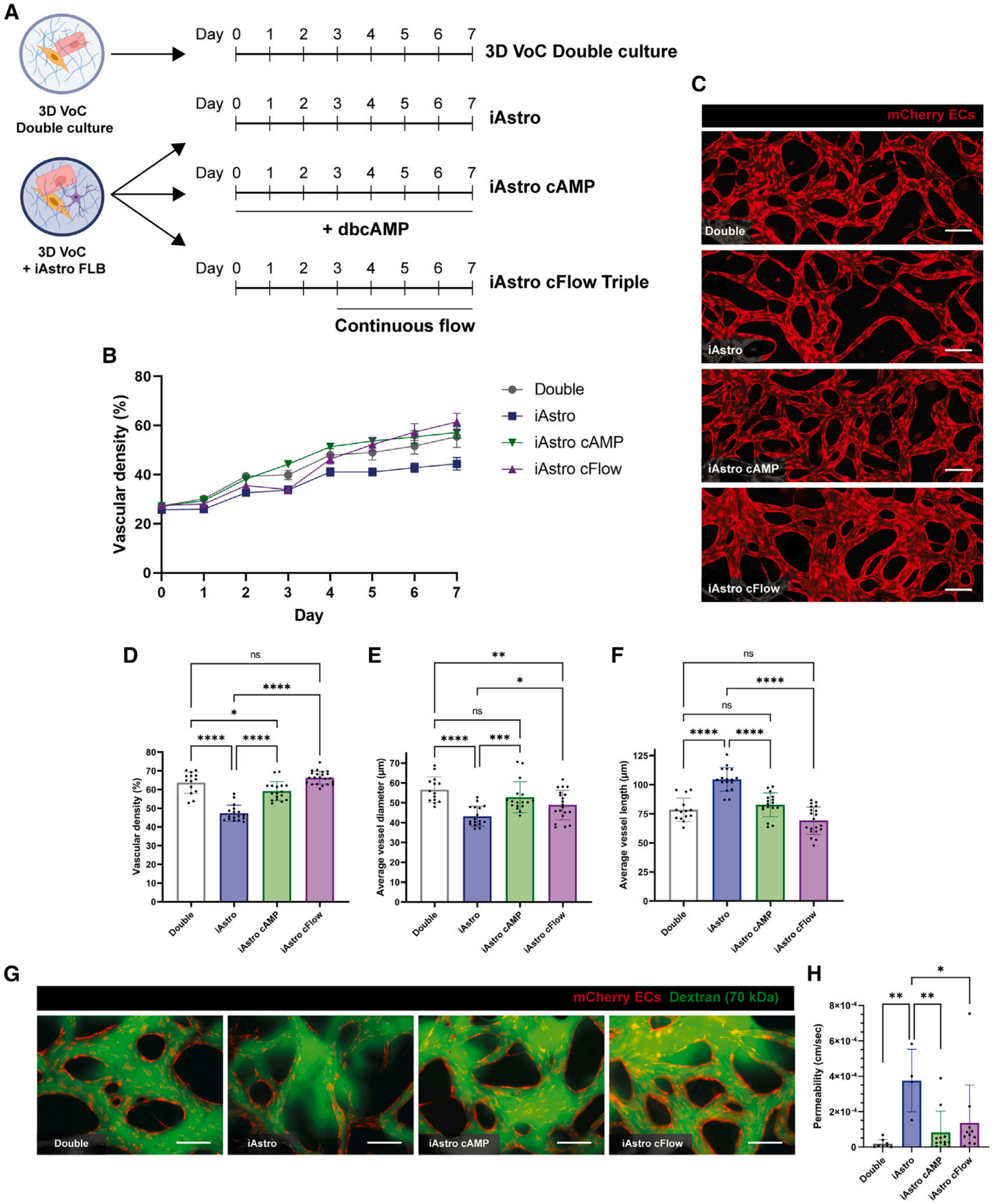

Figure 4. Improved microvascular network of 3D VoC triple culture including hiPSC-Astros upon activation of cAMP signaling or application of continuous perfusion

(A) Schematic of experimental setup for improving microvascular network formation of VoC triple cultures containing hiPSC-ECs, HBVPs, and iAstros from the FLB iPSC line. In the iAstro cAMP condition, the medium was daily supplemented with 250 μM dbcAMP to activate cAMP signaling. In the iAstro continuous flow (cFlow) condition, the VoC triple culture was subjected to continuous flow from day 3 onwards.

(B) Quantification of vessel density over time from daily images of hiPSC-mCherry ECs for the four VoC culture conditions.

(C) Representative immunofluorescence images of microvascular networks from the four VoC culture conditions at day 7 showing hiPSC-mCherry ECs (red). iAstro conditions are triple cultures containing iAstros from the FLB hiPSC line. Scale bars: 200 μm.

(legend continued on next page)

BBB-related genes investigated in triple culture conditions (Figure S4A). Integration of astrocytes had no effect on the expression of adherens and tight junction markers, such as vascular endothelial cadherin (VEC), zonula occludens-1 (ZO1), and claudin-5 (CLDN5) by real-time qPCR (Figure S4A) and by immunohistochemistry (Figure S4B). Some upregulation of transport-related genes, such as solute carrier family 2 member 1 (SLC2A1) and p-glycoproteïne (PGP), and extracellular matrix (ECM)-related gene collagen type IV alpha 1 chain (COL4A1) was observed by real-time qPCR (Figure S4A) under continuous flow, but this was less evident by immunohistochemistry (Figures S4C and S4D).

DISCUSSION

In this study, we described the generation of 3D microvascular networks containing hiPSC-ECs, HBVPs, and hiPSC-Astros. We showed that these triple cultures develop interconnected, lumenized, perfusable networks, with direct interaction between the incorporated cell types. No apparent differences were observed in the morphology and expression of reactive markers in astrocytes, in any of the culture conditions tested in this study. Although the number of HBVPs and their interaction with the microvascular network were similar between culture conditions, the average volume and expression of the contractile marker SM22 differed between HBVPs in double and triple cultures. This could indicate that both pAstros and hiPSC-Astros affect the maturity and contractile phenotype of the HBVPs in our system. Interestingly, we observed decreased vascular density and diameter of the vessels upon adding pAstros or iAstros, similar to earlier studies also using pAstros (Campisi et al., 2018; Lee et al., 2020). In addition, we showed that appropriate culture medium and fluidic flow are important in the formation and stability of microvascular networks containing hiPSC-ECs, HBVPs, and hiPSC-Astros. We demonstrated that the addition of dbcAMP improved microvascular network formation and organization and vascular permeability in the VoC model that included iAstros. This is in line with previous reports demonstrating the importance of cAMP signaling in astrocyte and EC function (Ishizaki et al., 2003; McRae et al., 2018; Reuschlein et al., 2019; Viña et al., 2021; Zhou et al., 2019). Interestingly, EC proliferation was not increased by adding dbcAMP, although we cannot exclude timing of the EdU experiment being sub-optimal. Future studies will need to clarify the exact mechanism by which dbcAMP acts in this 3D model. We also showed that microfluidic flow promoted EC proliferation and improved stability of the triple culture microvascular networks. In addition, we observed increased MMP2 expression as shown previously (Zhang et al., 2022), even though this earlier study investigated the effect of interstitial flow in the first stages of vasculogenesis while we only applied flow when a lumenized microvascular network had already formed, primarily resulting in luminal shear stress.

Although we observed interaction between various cell types in our system, adding iAstros to the VoC model did not consistently increase BBB-related markers in hiPSC-ECs. This was independent of whether iAstros were from FLB or TUBA hiPSC lines. Improvements in the metabolic environment, adding other relevant small molecules or cytokines or altering the transcriptional regulation of hiPSC-ECs using transcription factors, will be required for better recapitulation of the BBB (Lu et al., 2021). Nevertheless, the model does represent an opportunity to be entirely hiPSC based, by replacing HBVPs with hiPSC-derived smooth muscle cells (Vila Cuenca et al., 2021), which would allow investigation of cell type-specific contributions in disease phenotypes as each could be replaced by an (isogenic) mutant variant.

In summary, we established a 3D VoC model containing hiPSC-ECs, HBVPs, and hiPSC-Astros mimicking the direct cell-cell interactions seen *in vivo*. We demonstrated that hiPSC-Astros perform similarly to pAstros and can thus be used as an alternative to primary brain tissue. We demonstrated that our model can recapitulate the complex interactions between multiple cell types within the BBB, crucial for studying diseases like cerebral amyloid angiopathy, other forms of vascular dementia, and conditions related to neuroinflammation. However, we noted that the extent to which hiPSC-Astros influence microvascular network formation

(D–F) Quantification of microvascular networks showing vascular density at endpoint day 7 (D), average vessel diameter (E), and average vessel length (F). Data are shown as mean ± SD. Data shown are N = 3–4, n = 14–22; three or four independent experiments with a minimum of 3 microfluidic channels per experiment. iAstro conditions are triple cultures containing iAstros from the FLB hiPSC line.

(G) Representative immunofluorescence images of microvascular networks (red; hiPSC-mCherry ECs) perfused with 70 kDa FITC-dextran (green) on day 7, 30 s after start of perfusion. Images show VoC double cultures (hiPSC-ECs with HBVPs) and VoC triple cultures also containing iAstros from the FLB hiPSC line. Scale bars: 200 μm.

(H) Quantification of permeability coefficient for the four VoC culture conditions at endpoint day 7 from N = 3–4, n = 4–11; three or four independent experiments with one to six microfluidic channels per experiment. In the iAstro conditions, data are pulled from triple cultures containing both iAstros from the FLB and the TUBA hiPSC lines. One-way ANOVA with Sidak's multiple comparison test. *$p < 0.05$, **$p < 0.01$ ***$p < 0.001$, ****$p < 0.0001$; ns, non-significant. See also Figures S3 and S4.

can be hiPSC line dependent. Increasing cAMP signaling or introducing continuous flow improved microvascular networks containing hiPSC-Astros. Nevertheless, culture conditions will need further refinement before hiPSC models resemble the human BBB sufficiently for widespread use in studying neurodegenerative disorders and screening for new therapeutic interventions.

EXPERIMENTAL PROCEDURES

Resource availability

Lead contact

Requests for further information or more detailed protocols should be directed to and will be fulfilled by the corresponding author, Valeria V. Orlova (v.orlova@lumc.nl).

Materials availability

This study did not generate new unique reagents.

Data and code availability

Data will be shared with the research community upon request. No code or standardized datasets were generated.

hiPSC lines

Research on hiPSC was approved by the medical ethical committee at Leiden University Medical Center, the Netherlands. A detailed list of the hiPSC lines and batches used for each experiment is provided in Table S1.

VoC setup and culture

Cells were prepared prior to incorporation in VoCs as described in supplemental experimental procedures. Microvascular networks inside microfluidic chips were generated as previously described (Orlova et al., 2022; Vila Cuenca et al., 2021) with minor modifications. Microfluidic chips with one middle gel channel flanked by two media channels (AIM Biotech, idenTx9 chip) were used. Cells were resuspended in endothelial cell growth medium 2 (EGM-2) medium supplemented with thrombin (4 U/mL, Sigma, T4648) at 15 × 10^6 cells/mL for hiPSC-ECs, 3 × 10^6 cells/mL for HBVPs, and 7.5 × 10^6 cells/mL for astrocytes (5:1:2.5 ratio, respectively). Three astrocyte cell suspensions were tested in combination with hiPSC-ECs and HBVPs: (1) pAstros, (2) iAstros, and (3) iSCT Astros. The cell suspensions were mixed with an equal volume of fibrinogen solution (6 mg/mL, final concentration 3 mg/mL, Sigma, 8630) and immediately injected into the gel channel of the microfluidic chip (3 gel channels per cell/fibrin mix and 15 μL per gel channel). Chips were incubated for 15 min at room-temperature (RT) before adding EGM-2 supplemented with 50 ng/mL vascular endothelial growth factor (VEGF) and 1% astrocyte growth supplement (AGS; Sciencell, 1852) to the media channels. The microfluidic chips were refreshed every 24 h with EGM-2 supplemented with VEGF (50 ng/mL) and 1% AGS. Refreshing was done by application of a hydrostatic pressure over the medium channel by adding 100 μL medium to the right media ports and 50 μL medium to the left media ports. On day 1, γ-secretase inhibitor N-[N-(3,5-difluorophenacetyl)-*l*-alanyl]-*s*-phenylglycinet-butyl ester (DAPT, 10 μM, Sigma, D5942) was added for 24 h. For cAMP condition, the media were additionally supplemented with 250 μM dbcAMP (Sigma, D0627) for the entire duration of culture. For the continuous perfusion condition, the microfluidic chips were placed on an interval rocker platform (Perfusion Rocker, MIMETAS) set at a 5-degree inclination and 8 min cycle time from day 3 onwards.

Statistical analysis

Statistical analyses were performed using GraphPad Prism 9 software. Normality of the data was evaluated by the Shapiro-wilk test. One-way ANOVA with Tukey's multiple comparison test or Sidak's multiple comparison test was used for comparing multiple groups. Detailed statistics are indicated in each figure legend. The data are reported as mean ± SD.

SUPPLEMENTAL INFORMATION

Supplemental information can be found online at https://doi.org/10.1016/j.stemcr.2024.05.006.

ACKNOWLEDGMENTS

We thank the LUMC human iPSC Hotel for the generation and characterization of hiPSC lines and the LUMC confocal imaging facility (Lennard Voortman, Annelies Boonzaier – van der Laan) for help with imaging. Ruben van Helden is thanked for useful discussions and providing pipelines for analysis. Elga de Vries is thanked for providing several antibodies. Laurent Roybon is thanked for discussions and providing information on astrocyte differentiation. Ncardia is thanked for the use of the FDSS/μcell for the calcium experiments. The Allen Cell Collection, available from Coriell Institute for Medical Research, provided materials. Images were generated using Biorender.com. This work was supported by the Netherlands Organ-on-Chip Initiative, an NWO Gravitation project (024.003.001) funded by the Ministry of Education, Culture, and Science of the government of the Netherlands, and the Novo Nordisk Foundation Center for Stem Cell Medicine supported by Novo Nordisk Foundation grants (NNF21CC0073729).

AUTHOR CONTRIBUTIONS

Conceptualization, V.V.O.; methodology, D.M.N., M.V.C., and V.V.O.; software, D.M.N.; investigation, D.M.N., M.V.C., M.H., F.E.v.d.H., T.d.K., and J.-P.F.; visualization, D.M.N.; resources, C.L.M. and V.V.O.; writing – original draft, D.M.N., C.L.M., and V.V.O.; writing – review and editing, D.M.N., C.L.M., and V.V.O.; supervision, C.L.M. and V.V.O.; project administration, V.V.O.; funding acquisition, A.M.J.M.v.d.M., C.L.M., and V.V.O.

DECLARATION OF INTERESTS

The authors declare no competing interests.

Received: January 16, 2023
Revised: May 16, 2024
Accepted: May 16, 2024
Published: June 13, 2024

REFERENCES

Boyer-Di Ponio, J., El-Ayoubi, F., Glacial, F., Ganeshamoorthy, K., Driancourt, C., Godet, M., Perrière, N., Guillevic, O., Olivier Couraud, P., and Uzan, G. (2014). Instruction of circulating endothelial progenitors in vitro towards specialized blood-brain barrier and arterial phenotypes. PLoS One *9*, e84179. https://doi.org/10.1371/journal.pone.0084179.

Campinho, P., Vilfan, A., and Vermot, J. (2020). Blood Flow Forces in Shaping the Vascular System: A Focus on Endothelial Cell Behavior. Front. Physiol. *11*, 552. https://doi.org/10.3389/fphys.2020.00552.

Campisi, M., Shin, Y., Osaki, T., Hajal, C., Chiono, V., and Kamm, R.D. (2018). 3D self-organized microvascular model of the human blood-brain barrier with endothelial cells, pericytes and astrocytes. Biomaterials *180*, 117–129. https://doi.org/10.1016/j.biomaterials.2018.07.014.

Cecchelli, R., Aday, S., Sevin, E., Almeida, C., Culot, M., Dehouck, L., Coisne, C., Engelhardt, B., Dehouck, M.P., and Ferreira, L. (2014). A stable and reproducible human blood-brain barrier model derived from hematopoietic stem cells. PLoS One *9*, e99733. https://doi.org/10.1371/journal.pone.0099733.

Gastfriend, B.D., Nishihara, H., Canfield, S.G., Foreman, K.L., Engelhardt, B., Palecek, S.P., and Shusta, E.V. (2021). Wnt signaling mediates acquisition of blood–brain barrier properties in naïve endothelium derived from human pluripotent stem cells. Elife *10*, 1–33. https://doi.org/10.7554/eLife.70992.

Hajal, C., Le Roi, B., Kamm, R.D., and Maoz, B.M. (2021). Biology and Models of the Blood-Brain barrier. Annu. Rev. Biomed. Eng. *23*, 359–384.

Hajal, C., Offeddu, G.S., Shin, Y., Zhang, S., Morozova, O., Hickman, D., Knutson, C.G., and Kamm, R.D. (2022). Engineered human blood–brain barrier microfluidic model for vascular permeability analyses. Nat. Protoc. *17*, 95–128. https://doi.org/10.1038/s41596-021-00635-w.

Ishizaki, T., Chiba, H., Kojima, T., Fujibe, M., Soma, T., Miyajima, H., Nagasawa, K., Wada, I., and Sawada, N. (2003). Cyclic AMP induces phosphorylation of claudin-5 immunoprecipitates and expression of claudin-5 gene in blood-brain-barrier endothelial cells via protein kinase A-dependent and -independent pathways. Exp. Cell Res. *290*, 275–288. https://doi.org/10.1016/S0014-4827(03)00354-9.

Lee, S., Chung, M., Lee, S.R., and Jeon, N.L. (2020). 3D brain angiogenesis model to reconstitute functional human blood–brain barrier in vitro. Biotechnol. Bioeng. *117*, 748–762. https://doi.org/10.1002/bit.27224.

Lippmann, E.S., Azarin, S.M., Kay, J.E., Nessler, R.A., Wilson, H.K., Al-Ahmad, A., Palecek, S.P., and Shusta, E.V. (2012). Derivation of blood-brain barrier endothelial cells from human pluripotent stem cells. Nat. Biotechnol. *30*, 783–791. https://doi.org/10.1038/nbt.2247.

Lu, T.M., Houghton, S., Magdeldin, T., Durán, J.G.B., Minotti, A.P., Snead, A., Sproul, A., Nguyen, D.H.T., Xiang, J., Fine, H.A., et al. (2021). Pluripotent stem cell-derived epithelium misidentified as brain microvascular endothelium requires ETS factors to acquire vascular fate. Proc. Natl. Acad. Sci. USA *118*, e2016950118. https://doi.org/10.1073/pnas.2016950118.

Maoz, B.M., Herland, A., FitzGerald, E.A., Grevesse, T., Vidoudez, C., Pacheco, A.R., Sheehy, S.P., Park, T.-E., Dauth, S., Mannix, R., et al. (2018). A linked organ-on-chip model of the human neurovascular unit reveals the metabolic coupling of endothelial and neuronal cells. Nat. Biotechnol. *36*, 865–874. https://doi.org/10.1038/nbt.4226.

McRae, M., LaFratta, L.M., Nguyen, B.M., Paris, J.J., Hauser, K.F., and Conway, D.E. (2018). Characterization of cell-cell junction changes associated with the formation of a strong endothelial barrier. Tissue Barriers *6*, e1405774. https://doi.org/10.1080/21688370.2017.1405774.

Nishihara, H., Gastfriend, B.D., Soldati, S., Perriot, S., Mathias, A., Sano, Y., Shimizu, F., Gosselet, F., Kanda, T., Palecek, S.P., et al. (2020). Advancing human induced pluripotent stem cell-derived blood-brain barrier models for studying immune cell interactions. FASEB J. *34*, 16693–16715. https://doi.org/10.1096/fj.202001507RR.

Nishihara, H., Perriot, S., Gastfriend, B.D., Steinfort, M., Cibien, C., Soldati, S., Matsuo, K., Guimbal, S., Mathias, A., Palecek, S.P., et al. (2022). Intrinsic blood-brain barrier dysfunction contributes to multiple sclerosis pathogenesis. Brain *145*, 4334–4348. https://doi.org/10.1093/brain/awac019.

Orlova, V.V., Van Den Hil, F.E., Petrus-Reurer, S., Drabsch, Y., Ten Dijke, P., and Mummery, C.L. (2014). Generation, expansion and functional analysis of endothelial cells and pericytes derived from human pluripotent stem cells. Nat. Protoc. *9*, 1514–1531. https://doi.org/10.1038/nprot.2014.102.

Orlova, V.V., Nahon, D.M., Cochrane, A., Cao, X., Freund, C., van den Hil, F., Westermann, C.J.J., Snijder, R.J., Ploos van Amstel, J.K., Ten Dijke, P., et al. (2022). Vascular defects associated with hereditary hemorrhagic telangiectasia revealed in patient-derived isogenic iPSCs in 3D vessels on chip. Stem Cell Rep. *17*, 1536–1545. https://doi.org/10.1016/j.stemcr.2022.05.022.

Peteri, U.K., Pitkonen, J., Utami, K.H., Paavola, J., Roybon, L., Pouladi, M.A., and Castrén, M.L. (2021). Generation of the Human Pluripotent Stem-Cell-Derived Astrocyte Model with Forebrain Identity. Brain Sci. *11*, 209.

Reuschlein, A.K., Jakobsen, E., Mertz, C., and Bak, L.K. (2019). Aspects of astrocytic cAMP signaling with an emphasis on the putative power of compartmentalized signals in health and disease. Glia *67*, 1625–1636. https://doi.org/10.1002/glia.23622.

Roberts, B., Haupt, A., Tucker, A., Grancharova, T., Arakaki, J., Fuqua, M.A., Nelson, A., Hookway, C., Ludmann, S.A., Mueller, I.A., et al. (2017). Systematic gene tagging using CRISPR/Cas9 in human stem cells to illuminate cell organization. Mol. Biol. Cell *28*, 2854–2874. https://doi.org/10.1091/mbc.E17-03-0209.

Sweeney, M.D., Kisler, K., Montagne, A., Toga, A.W., and Zlokovic, B.V. (2018). The role of brain vasculature in neurodegenerative disorders. Nat. Neurosci. *21*, 1318–1331. https://doi.org/10.1038/s41593-018-0234-x.

Vatine, G.D., Barrile, R., Workman, M.J., Sances, S., Barriga, B.K., Rahnama, M., Barthakur, S., Kasendra, M., Lucchesi, C., Kerns, J., et al. (2019). Human iPSC-Derived Blood-Brain Barrier Chips Enable Disease Modeling and Personalized Medicine Applications.

Cell Stem Cell *24*, 995–1005.e6. https://doi.org/10.1016/j.stem.2019.05.011.

Vila Cuenca, M., Cochrane, A., van den Hil, F.E., de Vries, A.A.F., Lesnik Oberstein, S.A.J., Mummery, C.L., and Orlova, V.V. (2021). Engineered 3D vessel-on-chip using hiPSC-derived endothelial- and vascular smooth muscle cells. Stem Cell Rep. *16*, 2159–2168. https://doi.org/10.1016/j.stemcr.2021.08.003.

Viña, D., Seoane, N., Vasquez, E.C., and Campos-Toimil, M. (2021). cAMP compartmentalization in cerebrovascular endothelial cells: New therapeutic opportunities in alzheimer's disease. Cells *10*, 1–23. https://doi.org/10.3390/cells10081951.

Winkelman, M.A., Kim, D.Y., Kakarla, S., Grath, A., Silvia, N., and Dai, G. (2021). Interstitial flow enhances the formation, connectivity, and function of 3D brain microvascular networks generated within a microfluidic device. Lab Chip *22*, 170–192. https://doi.org/10.1039/d1lc00605c.

Zhang, S., Wan, Z., Pavlou, G., Zhong, A.X., Xu, L., and Kamm, R.D. (2022). Interstitial Flow Promotes the Formation of Functional Microvascular Networks In Vitro through Upregulation of Matrix Metalloproteinase-2. Adv. Funct. Mater. *32*, 2206767. https://doi.org/10.1002/adfm.202206767.

Zhou, Z., Ikegaya, Y., and Koyama, R. (2019). The astrocytic cAMP pathway in health and disease. Int. J. Mol. Sci. *20*, 779–827. https://doi.org/10.3390/ijms20030779.

Stem Cell Reports, Volume *19*

Supplemental Information

Self-assembling 3D vessel-on-chip model with hiPSC-derived astrocytes

Dennis M. Nahon, Marc Vila Cuenca, Francijna E. van den Hil, Michel Hu, Tessa de Korte, Jean-Philippe Frimat, Arn M.J.M. van den Maagdenberg, Christine L. Mummery, and Valeria V. Orlova

Inventory of Supplemental information

Supplemental figures and legends:
Figure S1. Related to Figure 1. Characterization of hiPSC-astrocytes.

Figure S2. Related to Figure 1. iSCT Astros incorporated into 3D VoC model.

Figure S3. Related to Figure 4. Microvascular network integrity in VoC conditions and increased proliferation and increased expression of MMP2 upon continuous flow in 3D VoC triple cultures including astrocytes.

Figure S4. Related to Figure 4. Assessment of blood-brain barrier properties in 3D VoC cultures.

Supplemental Table:
Supplemental Table 1. List of hiPSC lines and batches used per experiment.
Supplemental Table 2. List of antibodies for immunofluorescence.
Supplemental Table 3. List of primers for qRT-PCR.

Supplementary Video:
Video S1. Related to Figure 2 and Figure 3. 3D confocal reconstruction of EC-HBVP and EC-iAstro interactions

Supplemental Experimental Procedures

Supplemental References

SUPPLEMENTAL FIGURE 1.

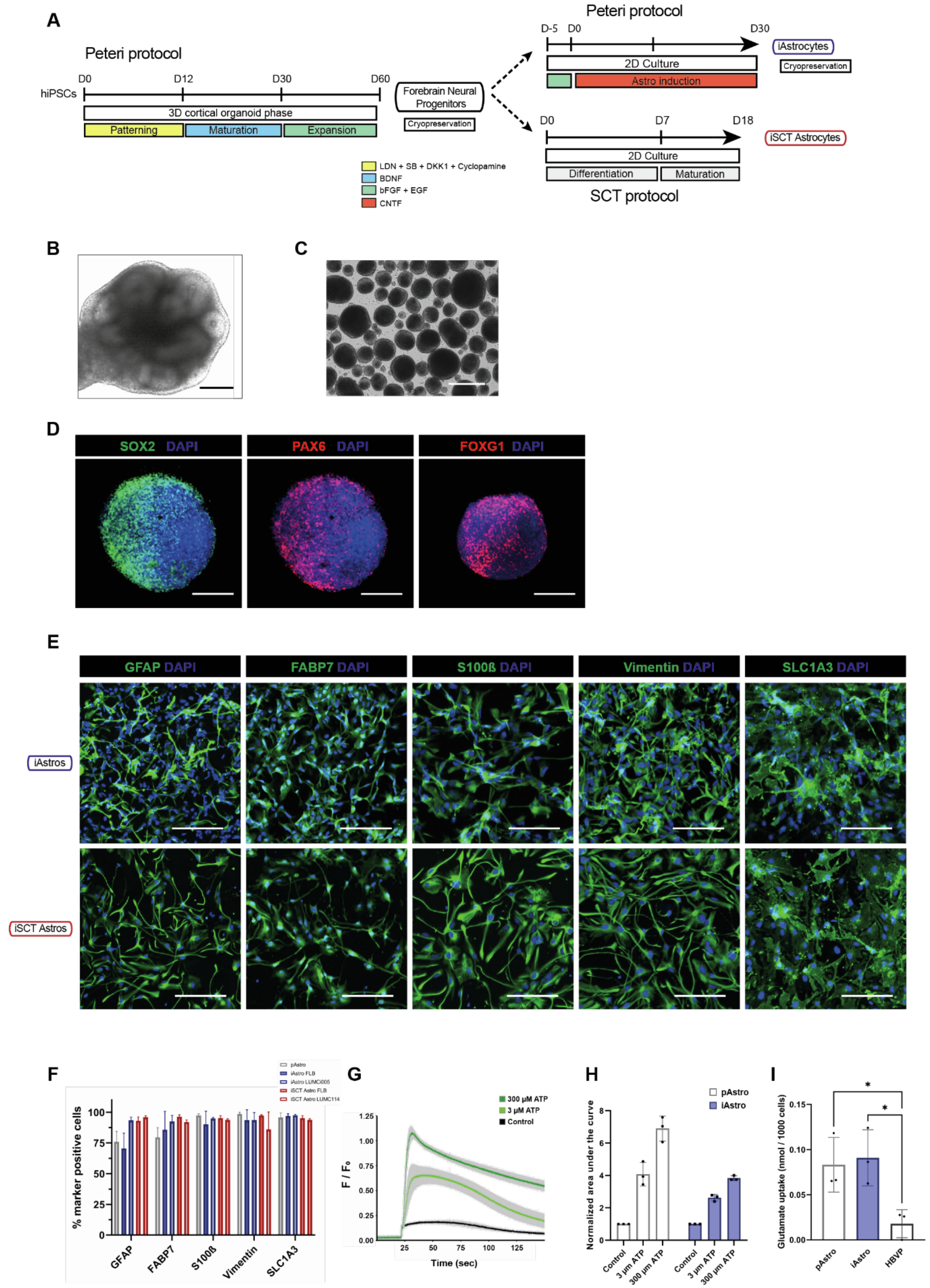

Figure S1. Related to Figure 1. Characterization of hiPSC-astrocytes.
(A) Schematic of 'Peteri' protocol (iAstro) and 'SCT' protocol (iSCT Astro) to generate forebrain patterned astrocytes from hiPSCs. (B) Representative brightfield image of a regionalized hiPSC-derived neural organoids on day 30. Scale bar: 250 µm. (C) Representative brightfield image of hiPSC-derived neural organoids on day 60. Scale bar: 500 µm. (D) Representative immunofluorescence images of hiPSC-derived neural organoids (day 72) differentiated from the FLB hiPSC line stained for SOX2, PAX6 or FOXG1. Scale bars: 100 µm. (E) Representative immunofluorescence images of iAstros and iSCT Astros stained for GFAP, FABP7, S100ß, Vimentin and GLAST. Scale bars; 200 µm. (F) Image based quantification of the percentage of marker positive cells. Data are shown as mean ± SD. pAstro, iAstro FLB and LUMCi005 are N = 3. iSCT Astro FLB and LUMC114 are N = 2. (G) Assessment of intracellular Ca^{2+} release in iAstros. Representative traces of normalized average fluorescence intensity (F/F0) in iAstros. Astrocytes were either stimulated by automated addition of plain NS medium (black) or NS medium supplemented with 3 µM (light green) or 300 µM (dark green) ATP. Data are shown as mean ± SD of N = 3; iAstros from one hiPSC line (FLB), one differentiation, in three independent experiments. (H) Quantification of intracellular Ca^{2+} release; normalized area under the curve, for pAstros and iAstros. Data shown as mean ± SD of N = 3; iAstros from one hiPSC line (FLB), three differentiations, in three independent experiments. (I) Quantified glutamate uptake normalized to the number of cells (nmol/1000 cells) for pAstros, iAstros and HBVP. Data shown as mean ± SD of N = 3, n = 6; one batch of pAstros and HBVPs and iAstros from one hiPSC line (FLB), three differentiations, in three independent experiments. One-way ANOVA with Tukey's multiple comparison. $*p < 0.05$; ns, non-significant.

SUPPLEMENTAL FIGURE 2.

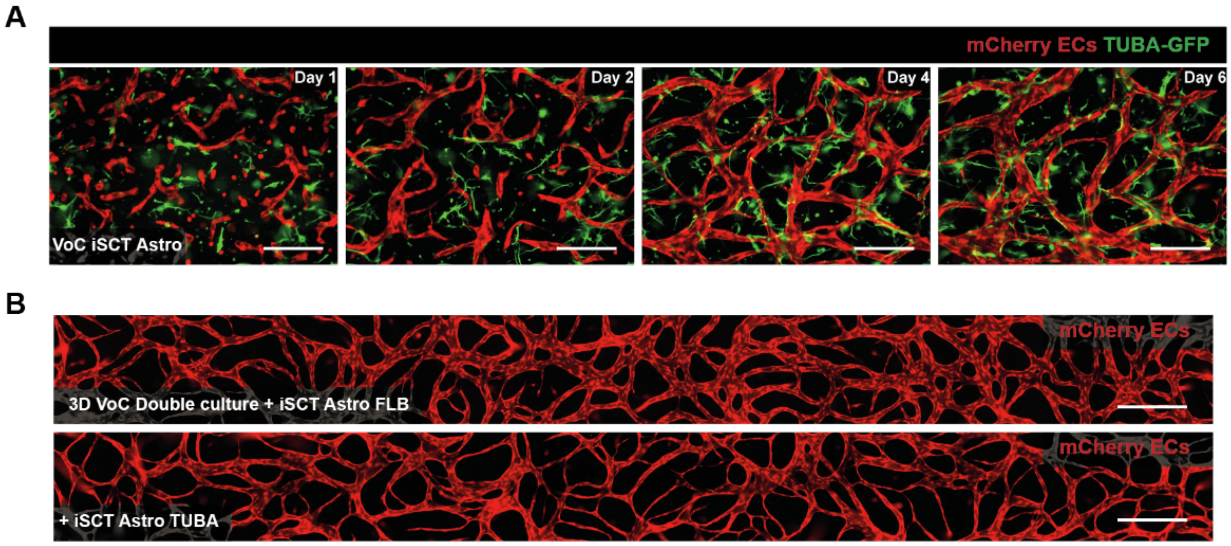

Figure S2. Related to Figure 1. iSCT Astros incorporated into 3D VoC model.
(A) Representative immunofluorescence images from day 1, 2, 4 and 6 showing hiPSC-mCherry ECs (red) and hiPSC-TUBA astrocytes (green, TUBA-GFP) from the 'SCT' protocol (iSCT Astro) in VoC triple cultures. Scale bars: 250 µm. (B) Representative immunofluorescence images of microvascular networks in microfluidic chips on day 7 showing hiPSC-mCherry ECs (red). Images showing microvascular networks from 3D VoC triple cultures including iSCT Astros from two independent hiPSC lines (FLB or TUBA). Scale bars: 250 µm.

SUPPLEMENTAL FIGURE 3.

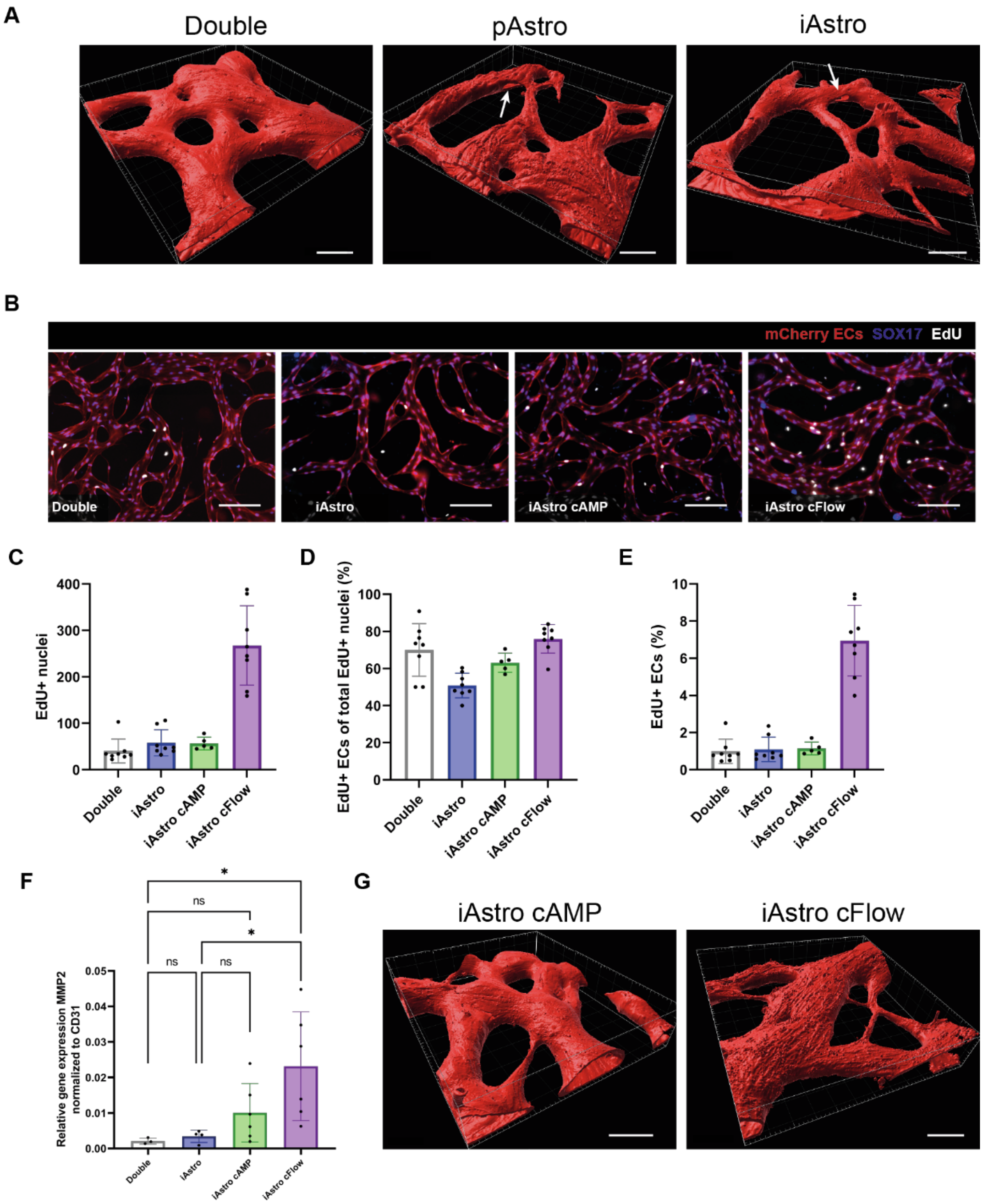

Figure S3. Related to Figure 4. Microvascular network integrity, increased proliferation and MMP2 expression upon continuous flow in 3D VoC triple cultures with astrocytes.

(A) Representative immunofluorescence confocal surface rendered images of microvascular networks in microfluidic chips of VoC cultures on day 7 showing hiPSC-mCherry ECs (red). Scale bars: 100 µm.

(B) Representative images showing proliferating cells (silver; EdU) and ECs (red and green; mCherry-ECs and SOX17 respectively) in VoC double cultures (hiPSC-ECs with HBVPs) and VoC triple cultures with iAstros from the FLB hiPSC line. In the iAstro cAMP condition, medium was daily supplemented with 250 µM dbcAMP and in iAstro continuous flow (cFlow) condition, microfluidic chips were continuously perfused from day 3 onwards. Microfluidic channels were fixed and stained at day 4. Scale bars: 200 µm. (C-E) Quantification of proliferation showing the total number of EdU positive nuclei (C), percentage of

proliferating cells which are ECs ((EdU+ SOX17+)/(EdU+)*100) (D) and percentage of ECs which are proliferating ((EdU+ SOX17+)/(SOX17+)*100) (E). Data shown as mean ± SD of N = 2, n = 8; two independent experiment with a minimum of 3 microfluidic channels per experiment. Exception is iAstro cAMP with N = 1, n = 5; one independent experiment with 5 microfluidic channels. (F) Relative expression of MMP2 normalized to CD31 as assessed with quantitative real-time PCR (qRT-PCR) for the four VoC culture conditions at end-point day 7. Data shown as mean ± SD from N = 3-6 independent experiments. In the iAstro conditions, data shown is from triple cultures containing both iAstros from the FLB and the TUBA hiPSC line. One-way ANOVA with Sidaks multiple comparison test. *$p < 0.05$ (G) Representative immunofluorescence confocal surface rendered images of microvascular networks in microfluidic chips of VoC cultures on day 7 showing hiPSC-mCherry ECs (red). Scale bars: 100 µm.

SUPPLEMENTAL FIGURE 4.

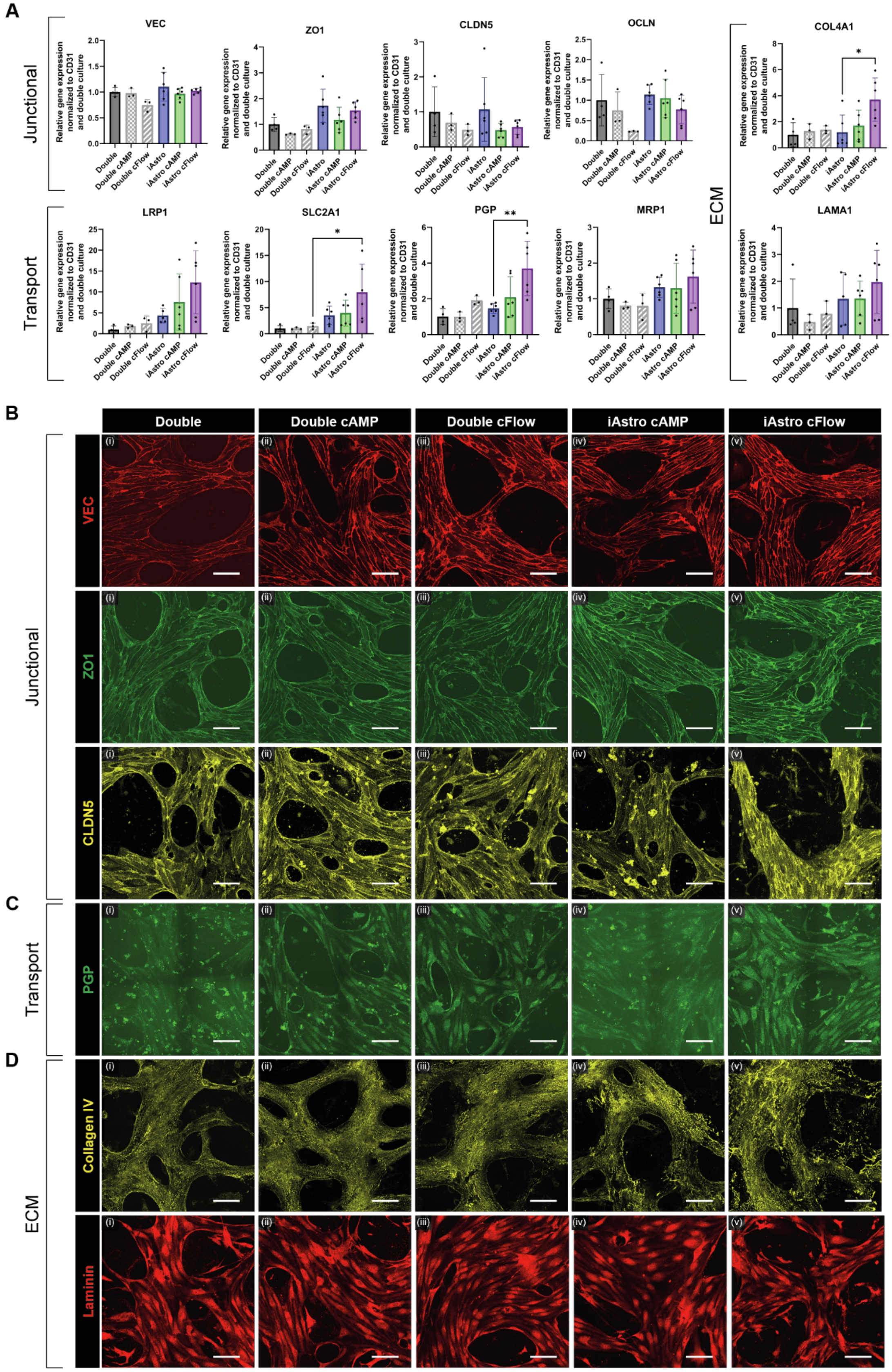

Figure S4. Related to Figure 4. Assessment of blood-brain barrier properties in 3D VoC cultures.
(A) RNA expression of key BBB-genes for the different 3D VoC culture conditions. Values were obtained with qRT-PCR and were normalized to housekeeping gene hARP and to CD31. Individual repeats are plotted for the conditions indicated. (B) Representative immunofluorescence confocal images for junctional proteins VE-Cadherin (VEC), Zonula occludens-1 (ZO1) and Claudin-5 (CLDN5). (C) Representative immunofluorescence confocal images for transporter protein P-glycoprotein (PGP). (D) Representative immunofluorescence confocal images for extracellular matrix (ECM) proteins Collagen IV and Laminin. Scale bars: 100 μm. Data are shown as mean ± SD. For N = 3-4, n = 3-6; three or four independent experiments with one pulled RNA sample per condition. In the iAstro conditions, data is pulled from triple cultures containing both iAstros from the FLB and the TUBA hiPSC lines. One-way ANOVA with Sidaks multiple comparison test. *$p < 0.05$, **$p < 0.01$; ns, non-significant.

Supplemental Table 1. List of hiPSC lines and batches used per experiment

Figure	hiPSC-ECs		hiPSC-Astros	
	Line	Number of batches	Line	Number of batches
Fig S1 F	-	-	iAstro: LUMC0020iCTRL	3
			iAstro: LUMCi005-A	1
			iSCT-Astro LUMC0020iCTRL	2
			iSCT-Astro LUMC114iCTRL01	2
Fig S1 H, I	-	-	LUMC0020iCTRL	3
Fig 1 D-F; 2 B, D, E	NCRM-1	3	LUMC0020iCTRL	2
			AICS-0012	1
Fig 3 C-F	NCRM-1	3	AICS-0012	1
Fig 4 D-F; S3 B-D	NCRM-1	3	LUMC0020iCTRL	2
Fig 4 G, I; S4 A	LUMC0054iCTRL02	1	LUMC0020iCTRL	1
			AICS-0012	1
Fig S4 C, E, G	LUMC0054iCTRL02	1	LUMC0020iCTRL	1

Supplemental Table 2. List of antibodies for IF

Antibody	Species	Source	Use	Dilution	Catalog #
SOX2	Rat	eBiosciences	2D	1:200	53-9811-80
PAX6	Rabbit	Cell Signaling	2D	1:200	60433S
FOXG1	Rabbit	Abcam	2D	1:200	ab18259
GFAP	Rabbit	DAKO	2D + 3D VoC	1:1500	Z033401
FABP7	Mouse	Santa Cruz	2D + 3D VoC	1:500	sc-374588
S100ß	Mouse	Sigma	2D	1:500	S2532
VIM	Mouse	Sigma	2D	1:300	V6630
SLC1A3/GLAST/EAAT1	Mouse	Miltenyi	2D	1:100	130-095-822
Aqp4	Rabbit	Novus Bio	3D VoC	1:200	NBP1-87679
NG2	Mouse	Santa Cruz	3D VoC	1:200	sc-53389
SM22/TAGLN	Rabbit	Abcam	3D VoC	1:400	ab14106
SOX17	Goat	R&D systems	3D VoC	1:300	AF1924
PGP	Mouse	Invitrogen	3D VoC	1:100	MA1-26528
Collagen IV	Goat	Millipore	3D VoC	1:200	AB769
Laminin	Rabbit	Sigma	3D VoC	1:100	AB19012

Supplemental Table 3. List of primers for qRT-PCR

Target	Forward primer (5' – 3')	Reverse primer (5' – 3')
VEC	GGCATCATCAAGCCCATGAA	TCATGTATCGGAGGTCGATGGT
CD31	GCATCGTGGTCAACATAACAGAA	GATGGAGCAGGACAGGTTCAG
ZO1	CAACATACAGTGACGCTTCACA	CACTATTGACGTTTCCCCACTC
CLDN5	GCGTGCTCTACCTGTTTTGC	CAGCTCGTACTTCTGCGACA
OCLN	ACAAGCGGTTTTATCCAGAGTC	GTCATCCACAGGCGAAGTTAAT
SLC2A1	AACTCTTCAGCCAGGGTCCAC	CACAGTGAAGATGATGAAGAC
PGP	TGACCCGCACTTCAGCTAC	GGGCTTCCCGATGATGTCG
MRP1	TTACTCATTCAGCTCGTCTTGTC	CAGGGATTAGGGTCGTGGAT
LRP1	CTATCGACGCCCCTAAGACTT	CATCGCTGGGCCTTACTCT
COL4A1	CAAAAGGGTGATACTGGAGAACC	ATTTCCTGCGAAACCAGGCA
LAMA1	GTGATGGCAACAGCGCAAA	GACCCAGTGATATTCTCTCCCA
MMP2	CTACGATGGAGGCGCTAATGG	CTTGGGGCAGCCATAGAAGG

Supplemental Experimental Procedures

hiPSC lines and maintenance

hiPSCs were maintained on recombinant vitronectin-coated plates in TeSR-E8, all from StemCell Technologies, according to the manufacturer's instructions. hiPSCs used for astrocyte differentiation were cultured on matrigel-coated (BD Biosciences, 354230) plates in TeSR^{TM}1 medium (StemCell Technologies, 05850) and mechanically passaged once a week using dispase solution 1 mg/mL (Gibco, 17105-041). The following hiPSC lines were used: LUMC0020iCTRL (Described in this report as FLB and generated from skin fibroblasts, https://hpscreg.eu/cell-line/LUMCi028-A) (Zhang et al., 2014). NIH Center for Regenerative Medicine hiPSC line (NCRM-1, generated from CD34+ cord blood cells, https://hpscreg.eu/cell-line/CRMi003-A), obtained from RUDCR Infinite Biologics at Rutgers University, was modified in-house with a mCherry expression cassette under the human cytomegalovirus (hCMV) early enhancer/chicken β actin (CAG) promoter using a previously established protocol (Rostovskaya et al., 2012). The Allen Cell Collection line AICS-0012 (Described in this report as TUBA and generated from skin fibroblasts, https://hpscreg.eu/cell-line/UCSFi001-A-2) with mEGFP insertion site at TUBA1B.

Differentiation of hiPSCs towards ECs

hiPSCs were maintained in mTeSR-E8 and differentiated towards ECs as previously described (Orlova et al., 2014b, 2014a). Briefly, mesoderm was induced by changing the media to B(P)EL medium supplemented with 8 µM CHIR99021 (Tocris Bioscience, 4423). Cells were refreshed at day 3, 6 and 9 with B(P)EL with VEGF (50 ng/mL) and 10 µM SB431542 (Tocris Bioscience, 1614). hiPSC-ECs were isolated on day 10 using CD31-Dynabeads™ (Thermo Fisher Scientific) as previously described (Orlova et al., 2014b, 2014a). hiPSC-ECs were expanded in complete EC growth medium comprised of Human Endothelial-serum free medium (EC-SFM) with 1% Human platelet poor serum (P2918, Sigma), VEGF (30 ng/mL) and bFGF (20 ng/mL). hiPSC-ECs were expanded for additional 3-4 days post-isolation and cryopreserved using serum-free cryopreservation medium at passage number 1 (P1) (CryoStor®CS10) (StemCell Technologies, 100-1061).

Differentiation of hiPSCs towards neural progenitors

Neural progenitor cells (NPCs) were generated through a regionalized neural organoid phase as described previously, with minor modifications (Peteri et al., 2021). Briefly, hiPSCs were dissociated using 0,5 mM Ethylenediaminetetraacetic acid (EDTA, Invitrogen, 15575020) and plated as small clumps 1:1 into ultra-

low attachment 6-well plate (Corning, 3471) or ultra-low T75 (Corning, 3814) in mTeSR-1 with 20 ng/mL bFGF (Miltenyi Biotec, 130-093-842) and RevitaCell (Life Technologies, 1:200). Following day an additional refreshment with mTESR-1, bFGF and Revitacell. The subsequent day neural induction and regional (forebrain) patterning was started by changing the medium to Neuronal Induction Medium (NIM) consisting of advanced DMEM/F12 (Life Technologies, 31331028), 2 mM L-glutamine (Life Technologies, 25030), 1% non-essential amino acids (NEAA; Life Technologies, 11140035), 1% N2 supplement (Life Technologies, 17502048), 1% Penicillin-Streptomycin (Life Technologies, 15070063) supplemented with 0.1 μM LDN-193189 (Axon Medchem), 10 μM SB-431542 (Tocris Bioscience, 1614), 0.5 μg/mL DKK-1 (PrepoTech, 120-30B) and 1 μM cyclopamine (R&D systems, 1623/1). Patterning took place from day 0 until day 12 with medium changes every second day. NPCs were matured from day 12 to day 30 by refreshment every second day with NIM medium supplemented with 20 ng/mL brain-derived neurotrophic factor (BDNF; Peprotech, 450-02). NPCs were expanded from day 30 by switching to Neurosphere (NS) Medium consisting of advanced DMEM/F12, 2 mM L-glutamine, 1% NEAA, 2% B27 supplement (Life Technologies, 17504044), 2 μg/mL heparin (Leo Pharma BV, 14179857) and 1% Penicillin-Streptomycin supplemented with 20 ng/mL bFGF and 20 ng/mL epidermal growth factor (EGF; R&D systems, 236-EG-200). Medium was changed two times a week and NPC spheres were manually dissociated to small clumps approximately every one and a half week. At day 60, NPC spheres were manually dissociated to small clumps and cryopreserved in 50 % NS medium, 40 % FBS (Biowest, S1860) and 10 % dimethyl sulfoxide (DMSO; Sigma, D2650). NPCs were thawed and cultured on 6-well plates coated with 20 μg/mL poly-ornithine (PO; Sigma, P3655) and 5 μg/mL laminin (Sigma, L2020) and maintained for 5 days in NS medium supplemented with 20 ng/mL bFGF and 20 ng/mL EGF to recover. The first day the media was also supplemented with 1:200 Revitacell. The NPCs were subsequently used for differentiation towards astrocytes.

Differentiation of neural progenitors towards astrocytes

NPCs were either differentiated using a previously published protocol (Peteri et al., 2021) (iAstros) or using a commercially available kit (iSCT Astros). In the iAstro differentiation, astrocyte specification was started by changing the media to NS medium supplemented with 20 ng/mL CNTF (Peprotech, 450-13). When reaching 80-90% confluency, cultures were passaged 1:4 using accutase (Millipore, SCR005). At day 30, astrocytes were cryopreserved in CryoStor®CS10. For characterization and functional assays of astrocytes, cryopreserved iAstros were thawed and cultured for 3 days on PO/laminin-coated plates in NS medium supplemented with 20 ng/mL CNTF.

For iSCT Astro differentiation, NPCs were passaged to matrigel-coated plates and maintained in STEMdiff™ Neural Progenitor Medium (StemCell Technologies, 05833) for 4-5 days to recover and expand. NPCs were subsequently differentiated using the STEMdiff™ Forebrain Neuron Differentiation Kit (StemCell Technologies, 08600), following manufacturer's protocol. After completing the 7 days differentiation protocol, medium was changed to BrainPhys™ Neuronal medium (StemCell Technologies, 05790) and cells were maintained for 11 more days before using in functional assays. Population of astrocytes was confirmed by positive staining for key astrocyte markers. Cells were passaged using accutase before use in microfluidic chips to enrich the astrocyte population.

Primary human brain vascular pericyte and primary astrocyte culture

Human brain vascular pericytes (HBVPs) and primary human cortical astrocytes (pAstros) were purchased from ScienceCell. HBVPs were cultured in Pericyte Medium (ScienceCell, 1201) supplemented with 1% Pericyte Growth Supplement (ScienceCell, 1252), 2% FBS and 1% penicillin/streptomycin. pAstros were cultured on poly-L-lysin coated (15 μg/mL, Sigma, P4707) plates in Astrocyte Medium (ScienceCell, 1801) supplemented with 1% Astrocyte Growth Supplement (AGS, Sciencell, 1852), 2% FBS and 1% penicillin/streptomycin. HBVPs and pAstros were cryopreserved at passage number 3 (P3) or 2 (P2) respectively, using serum-free cryopreservation medium (CryoStor®CS10) (StemCell Technologies, 100-1061).

Cell preparation prior to VoC culture

hiPSC-ECs (P1) were thawed and cultured on gelatin-coated plates in complete EC growth medium composed of Human Endothelial-SFM (EC-SFM) with 1% platelet poor serum (PPS), VEGF (30 ng/mL) and bFGF (20 ng/mL) 4 days prior to VoC seeding. HBVPs (P4) were thawed and cultured on gelatin-coated plates in Pericyte Medium (ScienceCell, 1201) supplemented with 1% Pericyte Growth Supplement

(ScienceCell, 1252), 2% FBS and 1% penicillin/streptomycin, 4 days prior to VoC seeding. pAstros were thawed and cultured in Astrocyte Medium (ScienceCell, 1801) supplemented with 1% Astrocyte Growth Supplement (AGS, Sciencell, 1852), 2% FBS and 1% penicillin/streptomycin, 4 days prior to VoC seeding. iAstros were thawed and cultured in NS medium supplemented with 20 ng/mL CNTF, 4 days prior to VoC seeding. iSCT-Astros were used directly after maturation at the end of the differentiation protocol. For an overview of the hiPSC lines and differentiation batches used for the different experiments, see Supplemental Table 1.

Immunostaining and microscopy of forebrain neural organoid
Forebrain neural organoids were fixed with 4% paraformaldehyde (PFA, Sigma) for 30 minutes at 4 °C and washed with phosphate-buffered saline (PBS) before continuing for wholemount staining. Cell plasma membranes were permeabilized with 0.5% Triton X-100 for 15 minutes at RT and washed 3 times for 10 minutes with PBS. Blocking was performed by adding 2% BSA in PBS for 3 hours at RT. Primary antibodies (see Supplemental Table 1 for details) were diluted in 1% BSA in PBS and incubated O/N at 4 °C. After washing with PBS, secondary antibodies (1:300, Invitrogen) diluted in 1% BSA were added and incubated for 2 hours at RT. Stained organoids were mounted with ProLong Gold Antifade Mountant (Thermofisher Scientific #P36930) on microscope slides. Images were taken using the EVOS M7000 using 20x magnification objective.

Immunostaining, microscopy and quantification of 2D astrocytes
iAstros were seeded on PO/laminin coated 96-well black imaging plates (Corning) at a seeding density of 35000 cells/well in NS medium supplemented with 20 ng/mL CNTF. iSCT-Astros were seeded on PO/laminin coated 96-well black imaging plates at a seeding density of 35000 cells/well in BrainPhys^{TM} Neuronal medium. Both were fixed 3 days later, using 4% PFA for 10 minutes at RT. Cell membranes were permeabilized with 0.1% Triton X-100 for 5 minutes at RT and washed with PBS before blocking with 1% BSA in PBS for 1 hour. Primary antibody diluted in 1% BSA were added and incubated O/N at 4 °C. For primary antibody overview see Supplemental table 1. Images were taken using EVOS M7000 using 10x magnification objective. Quantification of the percentage of marker positive astrocytes was performed using custom pipelines developed on the free open source CellProfiler software (https://cellprofiler.org/) (Carpenter et al., 2006). In brief, both nuclei and marker objects were identified after pre-processing steps to reduce unspecific object identification. Nuclei and marker objects were subsequently overlapped for visual examination of proper identification and to calculate number of masked nuclei objects.

Immunostaining and microscopy of VoC
Cells in VoCs were fixed *in situ* in 4% PFA for 30 minutes at RT. Cell plasma membranes were permeabilized with 0.5% Triton X-100 for 15 minutes at RT and washed 3 times for 10 minutes with PBS. Blocking was performed by adding 2% BSA in PBS for 3 hours at RT. Primary antibodies (see Supplemental Table 1 for details) were diluted in 1% BSA in PBS and incubated O/N at 4 °C. After washing with PBS, secondary antibodies (1:300, Invitrogen) diluted in 1% BSA were added and incubated for 2 hours at RT. Images of the full microfluidic channel of VoCs were taken with the EVOS M7000 using the 10x objective and automated stitching. 3D images were taken using the DragonFly spinning disk (Andor) microscope with 40x magnification objective, 2x2 tile scans with automated stitching and post-processing performed using Imaris 9.5 software (Bitplane, Oxford Instruments).

EdU assay for EC proliferation in 3D microfluidic chips
Proliferation was measured using the EdU Click-iT kit Alexa-488 (ThermoFisher Scientific #C10337) according to manufacturer's protocol. Briefly, on day 4 of culture, microfluidic chips were refreshed with EGM-2 supplemented with 50 ng/mL VEGF and 1% AGS additionally supplemented with EdU (1:1000) for 6 hours. Cells were fixed with 4% PFA for 30 minutes, permeabilized with 0.5% TX-100 for 15 minutes at RT. Freshly prepared Click-iT reaction cocktail was added for 3 hours at RT. Microfluidic chips were washed three times with PBS and blocked in 2% BSA in PBS for 3 hours at RT, followed by co-staining with primary and secondary antibodies.

Characterization of vascular and perivascular parameters in 2D images
Quantification of VoC vascular and perivascular parameters vessel density, average diameter, average vessel length, average astrocyte length and number, average HBVP length and number, EdU+ nuclei,

EdU+ ECs of total EdU+ nuclei, EdU+ ECs, PGP intensity and CollagenIV and Laminin area from 2D images was performed as previously described (Orlova et al., 2022; Vila Cuenca et al., 2021). Briefly, images of the whole microfluidic channel as acquired using EVOS M7000, were quantified using custom pipelines developed on the free open source CellProfiler software (https://cellprofiler.org/) (Carpenter et al., 2006). Pre-processing steps were applied to all images to enhance image features and a gaussian filter to reduce unspecific object identification. A minimum cross-entropy thresholding method was used on vascular network images to produce a binarized image. The binarized images from the CellProfiler output were then analyzed using ImageJ software with the freely available plugin DiameterJ (https://imagej.nih.gov/ij/, https://imagej.net/DiameterJ) (Hotaling et al., 2015). For quantification of CollagenIV and Laminin, a similar pipeline was used to generate binarized images. Quantification of PGP intensity was done by using a custom cell profiler pipeline with maximum projection images from 3D confocal images as input. Both intensity of PGP and area covered by either CollagenIV or Laminin staining was normalized by the area of the vessel, as determined by CD31 staining . EdU+ nuclei, EdU+ ECs of total EdU+ nuclei and EdU+ ECs were quantified with a custom-made pipeline in CellProfiler (Carpenter et al., 2006).

Characterization of astrocyte and HBVP cell parameters in 3D
Characterization and quantification of GFAP positive and SM22 positive objects and quantification of PGP intensity was performed by 3D quantitative analysis using images taken with the DragonFly spinning disk (Andor) and processed using Imaris 9.5 software (Bitplane, Oxford Instruments). Average HBVP SM22 object intensity and volume was obtained by first surface-rendering both individual SM22 positive objects and individual FABP7 or TUBA-GFP positive objects for the pAstro or iAstro conditions respectively. HBVP SM22 positive objects were obtained by filtering out the double positive objects. For quantification of the percentage of HBVP SM22 positive or GFAP positive objects touching the vessel, first an additional surface rendering of the microvascular network (mCherry positive) was performed. HBVP SM22 positive or GFAP positive objects touching the vessel was defined as a distance of 0 μm between the surface-rendered objects and >0 μm distance was defined as no contact.

Perfusion assessment in VoC system
Before the perfusion assessment and time-lapse imaging for permeability quantification, ECs were first stained using Ulex Europaeus Agglutinin I, DyLight594 (1:600, Vector Laboratories, DL-1067) by incubation of the microfluidic channels for 45 minutes in the incubator. Subsequently, time-lapse images were taken using the EVOS M7000 with on stage incubator with the 10x objective at 20 fps for 30 seconds. 70 μL of 70 KDa FITC-Dextran (1:1000, Sigma) in EGM-2 was added to one medium port and 50 μL of EGM- 2 to all other media ports to induce interstitial gravity driven flow. Agglutinin was imaged at the same location as the dextran perfusion was imaged to enable accurate assessment of fluorescent tracer leakage inside and outside the microvascular network. Calculation of permeability coefficient was based on previously established methods (Hajal et al., 2022). In short, the following formula was used:

$$P = \frac{1}{\Delta t}\frac{Am}{SPv}\frac{\Delta Im}{\Delta I}$$

With $\Delta t = T2 - T1$ (30 seconds), *Am* being the surface area of the matrix, *SPv* being the surface perimeter of the vessel, $\Delta Im = (Im2 * (Iv1/Iv2)) - Im1$ being the difference in fluorescence intensity in the matrix corrected for potential changes in the dextran concentration in the vascular space during imaging and $\Delta I = Iv1 - Im1$ being the difference in fluorescence intensity between the vasculature and matrix at the start of the measurement.

Assessment of intracellular Ca2+ release in astrocytes
Intracellular Ca^{2+} release was assessed in astrocytes at day one post-seeding in a black, flat-bottomed 96-well plate coated with poly-l-lysine for pAstros and PO/laminin for iAstros. The calcium-6 dye (Molecular Devices) was dissolved in 10 mL HBSS buffer B and subsequently diluted 1:4 in Buffer B (Molecular Devices). The diluted dye solution was added 1:1 to the wells containing astrocytes in NS medium. Astrocytes were incubated for 2 hours at 37 °C with 5% CO_2 before being measured on the FDSS/μcell (Hamamatsu Photonics) at 37 °C with an exposure time of 0.017s. Response to ATP stimulus was performed by first preparing a “compound plate” including a medium control of NS medium, 30 μM ATP (Sigma, A9187) in NS medium or 3 mM ATP in NS medium. Control and ATP stimulus from the “compound

plate" were automatically mixed and injected into the assay plate, reaching final ATP concentrations of 3 and 300 µM. Analysis was performed in R (4.0.3) and the induced change in Ca^{2+} release was calculated by quantification of the area under the curve of the average fluorescence intensity normalized to the NS medium control.

Glutamate uptake assay

A colorimetric glutamate assay kit (Sigma, MAK004) was used to determine the reduction of glutamate in the cell culture medium over time. Cells were plated 2 days before the assay in a 96 well plate. Before the assay, cells were washed with HBSS (Gibco) and then incubated with 100 µM glutamate in HBSS. Samples were collected and analyzed according to the manufacturer's instructions. The uptake of glutamate was normalized to the number of cells per well. Cells were stained with 1 µg/mL HOECHST 33342 (Thermo Fisher Scientific, 62249) for 20 minutes before washing with PBS. Whole wells were imaged using EVOS M7000 and subsequent downstream identification and quantification of the number of nuclei was performed with ImageJ software (https://imagej.nih.gov/ij/).

RNA isolation and quantitative RT-PCR

Total RNA was isolated from the microfluidic devices at end-point day 7. Cells were extracted by dissolving the extracellular matrix / fibrin mix with Collagenase B (1 mg/ml, Roche, 11088815001) for half an hour at 37 degrees °C, while rocking. RNA was extracted using the NucleoSpin RNA XS kit (Macherey-Nagel) and cDNA was synthesized using an iScript-cDNA Synthesis kit (Bio-Rad). iTaq Universal SYBR Green Supermixes (Bio-Rad) and Bio-Rad CFX384 real-time system were used for the PCR reaction and detection. Primers used can be found in Supplemental Table 3. Relative gene expression was calculated using the delta Ct calculation and normalized to the housekeeping gene hARP and to CD31. Heatmap was generated using the freely available online tool http://www.heatmapper.ca/expression/ (Babicki et al., 2016).

Supplemental References

Babicki, S., Arndt, D., Marcu, A., Liang, Y., Grant, J.R., Maciejewski, A., and Wishart, D.S. (2016). Heatmapper: web-enabled heat mapping for all. Nucleic Acids Res *44*, W147–W153. https://doi.org/10.1093/NAR/GKW419.

Carpenter, A.E., Jones, T.R., Lamprecht, M.R., Clarke, C., Kang, I.H., Friman, O., Guertin, D.A., Chang, J.H., Lindquist, R.A., Moffat, J., et al. (2006). CellProfiler: Image analysis software for identifying and quantifying cell phenotypes. Genome Biol *7*. https://doi.org/10.1186/gb-2006-7-10-r100.

Hajal, C., Offeddu, G.S., Shin, Y., Zhang, S., Morozova, O., Hickman, D., Knutson, C.G., and Kamm, R.D. (2022). Engineered human blood–brain barrier microfluidic model for vascular permeability analyses. Nat Protoc *17*, 95–128. https://doi.org/10.1038/s41596-021-00635-w.

Hotaling, N.A., Bharti, K., Kriel, H., and Simon, C.G. (2015). DiameterJ: A validated open source nanofiber diameter measurement tool. Biomaterials *61*, 327–338. https://doi.org/10.1016/j.biomaterials.2015.05.015.

Orlova, V. V., van den Hil, F.E., Petrus-Reurer, S., Drabsch, Y., ten Dijke, P., and Mummery, C.L. (2014a). Generation, expansion and functional analysis of endothelial cells and pericytes derived from human pluripotent stem cells. Nat Protoc *9*, 1514–1531. https://doi.org/10.1038/nprot.2014.102.

Orlova, V. V., Drabsch, Y., Freund, C., Petrus-Reurer, S., Van Den Hil, F.E., Muenthaisong, S., Ten Dijke, P., and Mummery, C.L. (2014b). Functionality of endothelial cells and pericytes from human pluripotent stem cells demonstrated in cultured vascular plexus and zebrafish xenografts. Arterioscler Thromb Vasc Biol *34*, 177–186. https://doi.org/10.1161/ATVBAHA.113.302598.

Orlova, V. V., Nahon, D.M., Cochrane, A., Cao, X., Freund, C., van den Hil, F., Westermann, C.J.J., Snijder, R.J., Ploos van Amstel, J.K., ten Dijke, P., et al. (2022). Vascular defects associated with

hereditary hemorrhagic telangiectasia revealed in patient-derived isogenic iPSCs in 3D vessels on chip. Stem Cell Reports *17*, 1536–1545. https://doi.org/10.1016/j.stemcr.2022.05.022.

Peteri, U., Pitkonen, J., Utami, K.H., Paavola, J., Roybon, L., Pouladi, M.A., and Castren, M.L. (2021). Generation of the Human Pluripotent Stem-Cell-Derived Astrocyte Model with Forebrain Identity. Brain Sci *11*.

Rostovskaya, M., Fu, J., Obst, M., Baer, I., Weidlich, S., Wang, H., Smith, A.J.H., Anastassiadis, K., and Francis Stewart, A. (2012). Transposon-mediated BAC transgenesis in human ES cells. Nucleic Acids Res *40*. https://doi.org/10.1093/nar/gks643.

Vila Cuenca, M., Cochrane, A., van den Hil, F.E., de Vries, A.A.F., Lesnik Oberstein, S.A.J., Mummery, C.L., and Orlova, V. V. (2021). Engineered 3D vessel-on-chip using hiPSC-derived endothelial- and vascular smooth muscle cells. Stem Cell Reports *16*. https://doi.org/10.1016/j.stemcr.2021.08.003.

Zhang, M., D’Aniello, C., Verkerk, A.O., Wrobel, E., Frank, S., Ward-Van Oostwaard, D., Piccini, I., Freund, C., Rao, J., Seebohm, G., et al. (2014). Recessive cardiac phenotypes in induced pluripotent stem cell models of Jervell and Lange-Nielsen syndrome: Disease mechanisms and pharmacological rescue. Proc Natl Acad Sci U S A *111*, E5383–E5392. https://doi.org/10.1073/pnas.1419553111.
